# Synthesis, biological evaluation and molecular docking investigation of new sulphonamide derivatives bearing naphthalene moiety as potent tubulin polymerisation inhibitors

**DOI:** 10.1080/14756366.2021.1943378

**Published:** 2021-06-22

**Authors:** Guangcheng Wang, Meiyan Fan, Wenjing Liu, Min He, Yongjun Li, Zhiyun Peng

**Affiliations:** a State Key Laboratory of Functions and Applications of Medicinal Plants, Guizhou Provincial Key Laboratory of Pharmaceutics, Guizhou Medical University, Guiyang, China; b School of Pharmacy, Guizhou Medical University, Guiyang, China; c Engineering Research Center for the Development and Application of Ethnic Medicine and TCM (Ministry of Education), Guizhou Medical University, Guiyang, China; d College of Food Science and Technology, Shanghai Ocean University, Shanghai, China

**Keywords:** Sulphonamide, trimethoxyphenyl, tubulin polymerisation inhibitors, anticancer activity

## Abstract

A new series of sulphonamide derivatives bearing naphthalene moiety were synthesised and evaluated for their antiproliferative and tubulin polymerisation inhibitory activities. These new compounds were evaluated for their *in vitro* antiproliferative activity against MCF-7 and A549 by using CCK-8 method. Among all the tested compounds, compound **5c** with naphthalen-1-yl moiety exhibited the most potent antiproliferative activity against MCF-7 and A549 cell line, with IC_50_ values of 0.51 ± 0.03 µM and 0.33 ± 0.01 µM, respectively. The results of tubulin polymerisation assay shown that **5c** exhibited a significant ability to inhibit tubulin polymerisation with IC_50_ value of 2.8 μM. Consistent with its antitubulin activity, **5c** can significantly arrest the cell cycle at G2/M phase and induce apoptosis in MCF-7 cancer cells. Molecular docking study indicated that compound **5c** inhibited tubulin polymerisation through interacting at the colchicine-binding site of tubulin. Furthermore, **5c** exhibited low cytotoxic activity on human normal cell line.

## 1. Introduction

Microtubules are crucial elements of the cytoskeleton in eukaryocyte, which are polymerised by *α*- and *β*-tubulin heterodimers in a head-to-tail manner to form hollow cylindrical filaments^1^. The microtubule system of eukaryotic cells plays important roles in numerous essential cellular functions, such as cell growth, division, motility, maintenance of cell shape, and intracellular vesicle transport ^2^^,^^3^. There is an increasing evidence showing that the disruption of microtubule will result in the cycle arrest in G2/M phase and lead to the apoptosis of cell ^4^. Therefore, microtubule has become an attractive molecular target in anti-tumour drug discovery ^5–7^. Up to the present, a larger number of anti-tubulin agents have been developed and some of them (e.g., paclitaxel, docetaxel, vinblastine, and vincristine) have been approved by FDA for clinical treatment of cancer ^8^. However, these natural products and their derivatives are facing severe disadvantages, such as toxicity, drug resistance, and difficult synthesis ^9^. Therefore, the search of novel molecules with potent tubulin polymerisation inhibitory activity is still in progress.

**Figure 1.**
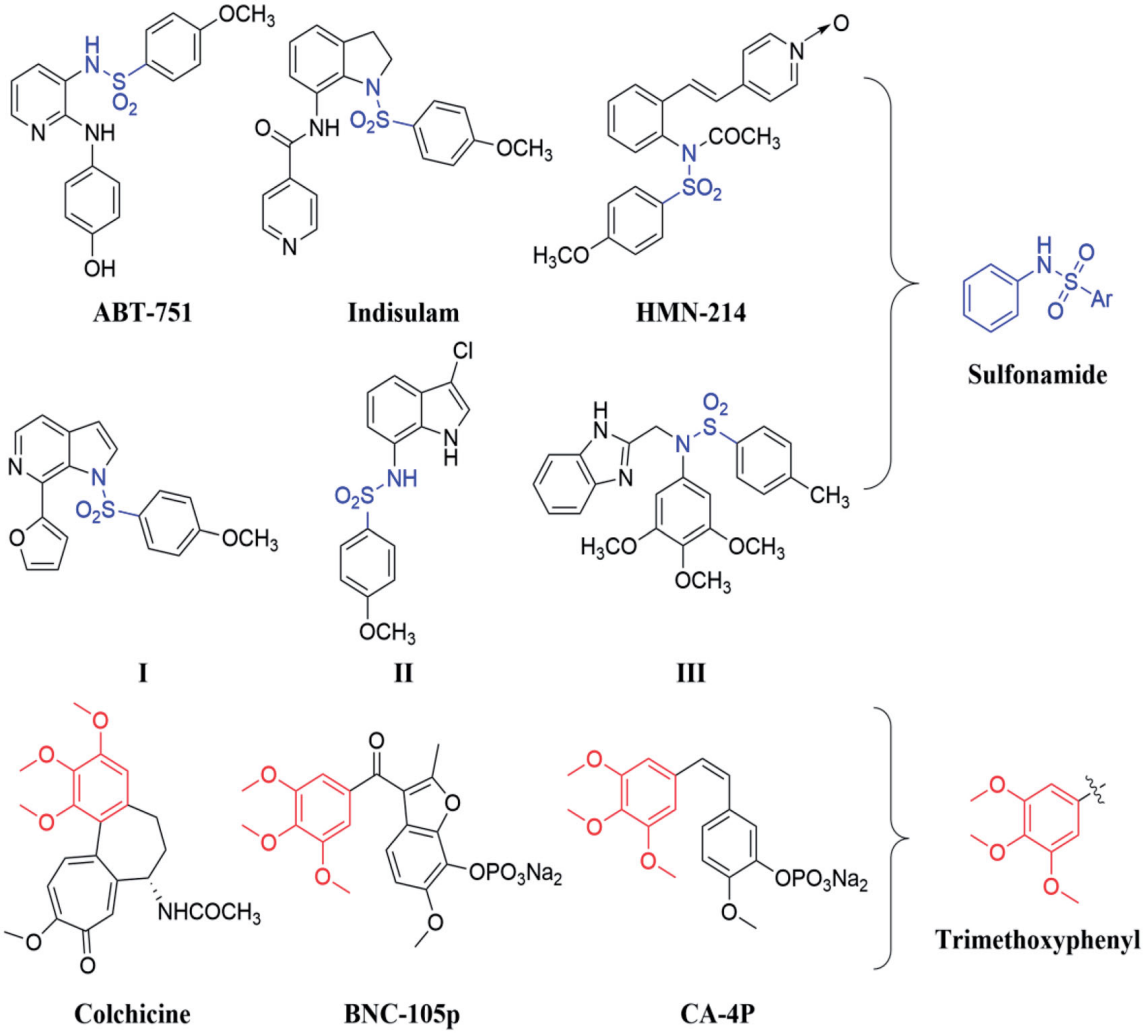
Chemical structures of some tubulin polymerisation inhibitors containing sulphonamide or trimethoxyphenyl moiety.

In recent years, sulphonamide and its derivatives have attracted much attention for their versatile properties in medicinal chemistry and modern drug discovery, such as anticancer, antiviral, antifungal, anti-inflammatory, antimicrobial, antibacterial, anti-tuberculosis, antiparasitic, anticonvulsant, carbonic anhydrase inhibition, and antidepressant activities ^10^. Notably, several compounds containing sulphonamide moiety have been reported inhibit tubulin polymerisation, such as **ABT-751**, **indisulam**, **HMN-214**, **I**, **III**, and **III** (Figure 1)^11–15^. These previous literatures reveal that sulphonamide could be used as a useful pharmacophoric fragment for the design and development anti-tubulin agents.

On the other hand, the trimethoxyphenyl has been proved to be an important pharmacophoric group of tubulin inhibitors by binding at the colchicine binding site of tubulin ^16^. Based on this, the trimethoxyphenyl moiety has been chosen as a core for the design and development of novel tubulin polymerisation inhibitors ^16^. In the last few decades, a large number of tubulin polymerisation inhibitors containing trimethoxyphenyl moiety have been reported in the literature, and some of them have entered clinical trials, such as **BNC-105p**, **CA-4P**, **CKD-516**, and **AVE8062** (Figure 1) ^17–20^.

**Figure 2.**
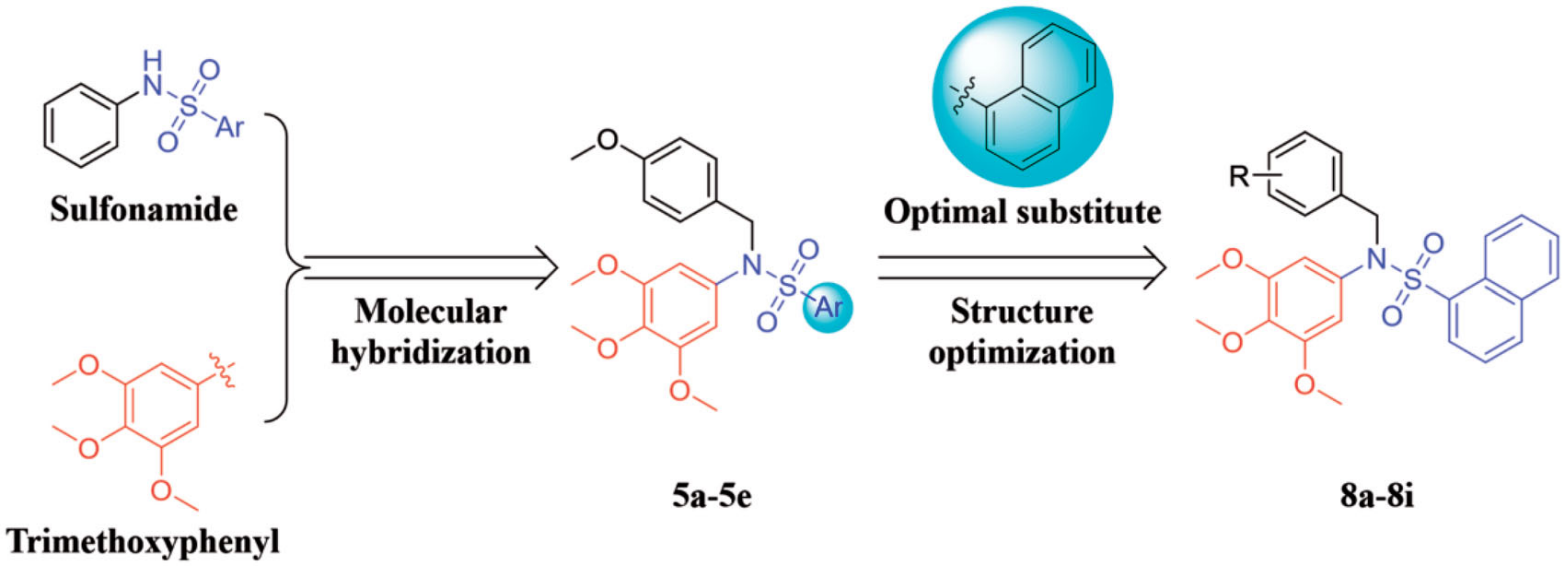
The design strategy of the target compounds in this study.

Hence, in continuation of our interest on the design and development of novel tubulin polymerisation inhibitors ^21–24^, a new series of sulphonamide derivatives (**5a**-**5e** and **8a**-**8i**) were designed based on the molecular hybridisation approach (Figure 2) ^25^^,^^26^. All the newly synthesised target compounds were evaluated for their antiproliferative activity to explore the preliminary structure-activity relationships (SAR). Tubulin polymerisation inhibition assay, cell cycle analysis, and cell apoptosis assay were performed to illuminate the pharmacologic mechanism. Additionally, molecular modelling was carried out to elucidate its possible binding mode in tubulin.

## 2. Results and discussion

### 2.1. Chemistry

**Scheme 1.**
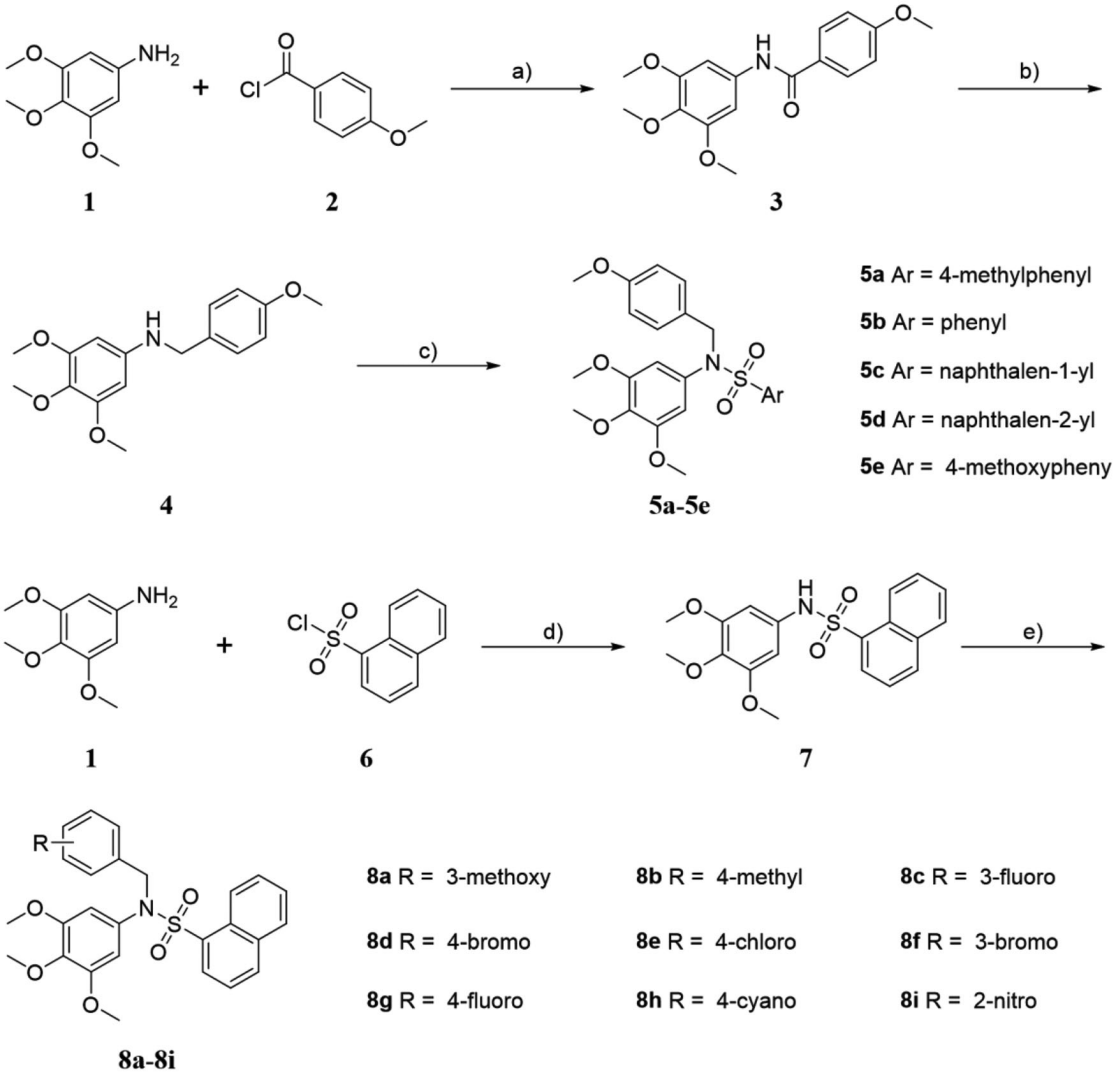
(a) Et_3_N, THF, room temperature, 4 h; (b) LiAlH_4_, THF, 0 °C 30 min to reflux, 2 h; (c) various aryl sulphonyl chloride, DMAP, Et_3_N, THF, room temperature, 12 h; (d) Et_3_N, CH_2_Cl_2_, room temperature, 6 h; (e) various benzyl halide, KI, K_2_CO_3_, acetone, reflux, 5 h.

The sulphonamide derivatives (**5a**-**5e** and **8a**-**8i**) were synthesised according to the synthetic route illustrated in Scheme 1. Firstly, 3,4,5-trimethoxyaniline **1** was condensed with 4-methoxybenzoyl chloride **2** in the presence of Et_3_N as base at room temperature to afford intermediate **3** in high yields, followed by the carbonyl reduction reaction with LiAlH_4_ to give the key intermediate **4** ^[^^27^^]^. Then, condensation of compound **4** with appropriate commercially available aryl sulphonyl chloride in the presence of Et_3_N and DMAP in THF to generate the title compounds (**5a**-**5e**) in high yields. On the other hand, treatment of 3,4,5-trimethoxyaniline **1** with naphthalene-1-sulphonyl chloride **6** in the presence of Et_3_N in CH_2_Cl_2_ to afforded key intermediate **7**, which reacted with commercially available benzyl halide in the presence of KI and K_2_CO_3_ to provide the title compounds (**8a**-**8i**). All target derivatives were fully characterised by ^1^H NMR, ^13 ^C NMR, HRMS, and elemental analysis (see Supporting Information).

**Figure 3.**
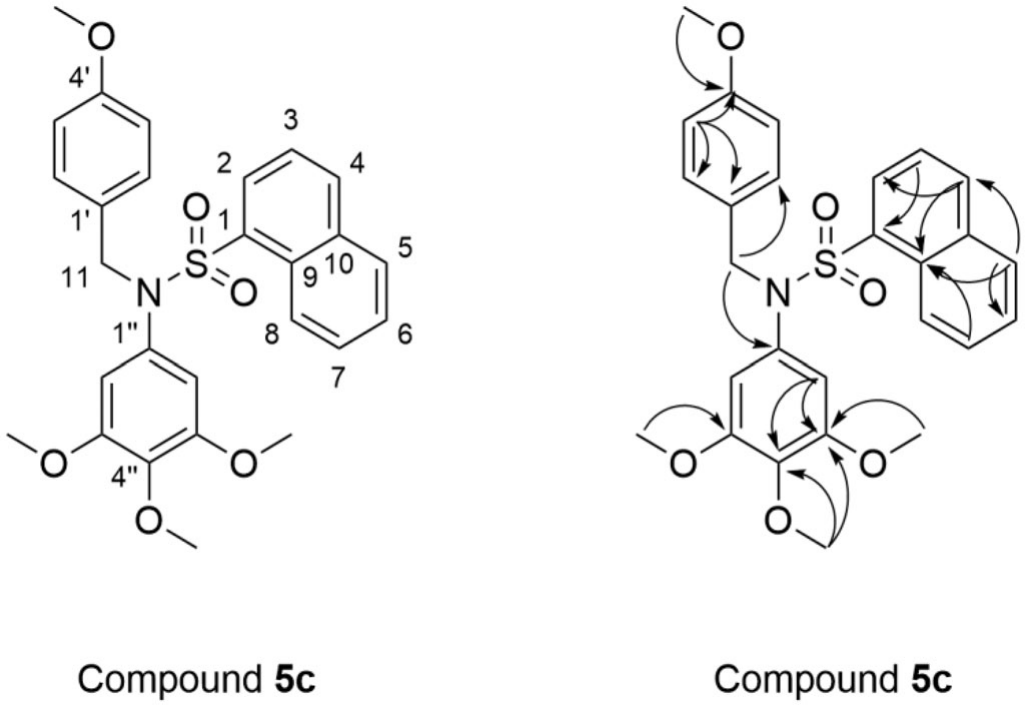
Chemical structure and key HMBC correlations of compound **5c**.

The molecular formula of compound **5c** was determined to be C_27_H_27_NO_6_S by high-resolution mass spectrum (HRMS) peak at m/z 516.1432 as [M + Na]^+^. The ^1^H NMR spectrum (Table S1 and Figure 3) of **5c** displayed four singlets at *δ* 3.33 (6H, s, OCH_3_-C-3′′, C-5′′), 3.53 (3H, s, OCH_3_-C-4′′), and 3.63 (3H, s, OCH_3_-C-4′) ppm due to four methoxy groups on the phenyl rings. The methylene protons of –CH_2_– was appeared as singlet at *δ* 4.69 ppm (s, 2H, H-11). Two doublet peaks at *δ* 6.75 and *δ* 7.09 ppm with coupling constant of 8.4 Hz were attributed to the C-3′,5′-H and C-2′,6′-H of 4-methoxybenzyl moiety, respectively. According to the aromatic proton signal at at *δ* 6.11 ppm (s, 2H, H-2′′, 6′′), there was a 3,4,5-trimethoxyphenyl in the structure. Seven aromatic hydrogen atoms of naphthalen-1-yl were appeared at *δ* 7.46–8.26 ppm (7H, H-2, 3, 4, 5, 6, 7, and 8). In ^13 ^C NMR spectrum of **5c** the signal at *δ* 53.37 ppm (C-11) was assigned to the carbon in methylene. The signals at *δ* 55.50 (OCH_3_-C-4′), 56.20 (OCH_3_-C-3′′, 5′′), and 60.61 (OCH_3_-C-4′′) ppm were attributed to the carbon in four methoxyl groups. The carbon of –CH_2_– was appeared at *δ* 53.37 ppm (C-11). In addition, there are 17 carbon signals at *δ* 107.41 (C-2′′, 6′′), 114.18 (C-3′, 5′), 125.24 (C-3), 125.36 (C-8), 127.40 (C-6), 128.15 (C-7), 128.59 (C-1′), 128.83 (C-9), 129.35 (C-5), 130.24 (C-2′, 6′), 131.17 (C-2), 133.95 (C-1), 134.08 (C-1′′), 134.32 (C-10), 135.17 (C-4), 137.59 (C-4′′), 152.80 (C-3′′, 5′′), 159.10 (C-4′).

The HMBC correlations from the aromatic proton at *δ* 8.05 (H-5) to C-6, C-9 and C-4, from *δ* 7.65 (H-3) to C-1 and C-10, from *δ* 7.48 (H-7) to C-9 indicated the presence of a 1-naphthalene sulphonyl moiety. In addition, the correlations from the methoxy proton at *δ* 3.63 (H-OCH_3_-C-4′) to C-4′, from *δ* 3.33 (H-OCH_3_-C-3′′, 5′′) to C-3′′ and 5′′, from *δ* 3.53 (H-OCH_3_-C-4′′) to C-4′′ and C-3′′ showed four methoxy substituents connected to C-4′, C-3′′, 5′′, and C-4′′, respectively. Furthermore, key HMBC correlations between *δ* 4.69 (H-11) and C-2′, C-6′ and C-1′′ showed that 4-methoxybenzyl and 3,4,5-trimethoxyphenyl were linked through N. Therefore, the spectral data were in full agreement with the expected structure of the compound **5c** (Figure 3 and Table S2).

### 2.2. *In vitro* antiproliferative activities and SARs

The *in vitro* antiproliferative activities of these sulphonamide derivatives (**5a**-**5e** and **8a**-**8i**) were evaluated against MCF-7 human breast cancer cells and A549 human non-small cell lung carcinoma cells by using CCK-8 method. Cisplatin, 5-fluorouracil (5-Fu), tamoxifen, and combretastatin A-4 (CA-4) were used as positive control. The results expressed as IC_50_ (µM) were summarised in Table 1. Among the series, compounds **5a**, **5c**, **5e**, and **8 b** showed potent anticancer activity against both MCF-7 and A549 cell lines with IC_50_ between 0.33 ± 0.01 and 5.34 ± 0.31 µM. All these compounds were more potent than cisplatin (IC_50_ = 11.15 ± 0.75 µM), 5-Fu (IC_50_ = 11.61 ± 0.60 µM), tamoxifen (IC_50_ = 14.28 ± 0.40 µM), and CA-4 (IC_50_ = 5.55 ± 0.11 µM) on MCF-7 cancer cell line. In particular, compound **5c** with a methoxyl group at the para position of phenyl ring and naphthalen-1-yl at the sulphonamide exhibited the most potent anticancer activity against MCF-7 and A549 cell lines (IC_50_ = 0.51 ± 0.03 and 0.33 ± 0.01 µM, respectively).

**Table 1.** *In vitro* cell growth inhibitory effects of compounds (**5a**-**5e** and **8a**-**8i**).

Compound	Ar/R	IC_50_ (μM)^a^	
		MCF-7	A549
**5a**	Ar = 4-methylphenyl	3.64 ± 0.25	5.34 ± 0.31
**5b**	Ar = phenyl	>30.0	>30.0
**5c**	Ar = naphthalen-1-yl	0.51 ± 0.03	0.33 ± 0.01
**5d**	Ar = naphthalen-2-yl	5.72 ± 0.18	>30.0
**5e**	Ar = 4-methoxypheny	0.87 ± 0.05	0.62 ± 0.04
**8a**	R = 3-methoxy	19.98 ± 0.71	>30.0
**8b**	R = 4-methyl	2.20 ± 0.19	1.29 ± 0.19
**8c**	R = 3-fluoro	>30.0	>30.0
**8d**	R = 4-bromo	8.02 ± 0.58	>30.0
**8e**	R = 4-chloro	15.59 ± 0.34	14.39 ± 0.80
**8f**	R = 3-bromo	>30.0	>30.0
**8g**	R = 4-fluoro	>30.0	>30.0
**8h**	R = 4-cyano	>30.0	>30.0
**8i**	R = 2-nitro	>30.0	4.76 ± 0.31
**Cisplatin**		11.15 ± 0.75	4.92 ± 0.46
**5-Fu**		11.61 ± 0.60	2.75 ± 0.31
**Tamoxifen**		14.28 ± 0.40	20.20 ± 0.65
**CA-4**		5.55 ± 0.11	0.029 ± 0.004

^a^ The values given are means of three experiments.

To study the structure-activity relationships (SAR) of this class of compounds, the aryl substituents on sulphonamides were discussed firstly. Based on the antiproliferative activity of compounds **5a**-**5e**, it can be seen that the aryl substituents on sulphonamides affected on antiproliferative activity of this class of compounds. Among these molecules, **5 b** with a phenyl ring displayed low antiproliferative activity with IC_50_ value of > 30.0 µM. Introduction of electron-donating (**5a** and **5e**) groups into the phenyl ring, results in significantly increased the antiproliferative activity. The replacement of phenyl ring with naphthalen-1-yl or naphthalen-2-yl led to compounds **5c** and **5d**, resulting in significantly increased the antiproliferative activity. In particular, compound **5c** with naphthalen-1-yl moiety was found to be the most active compound in this series. These inhibitory results indicate that naphthalen-1-yl group seems to be the optimal substituent on the position.

**Figure 4.**
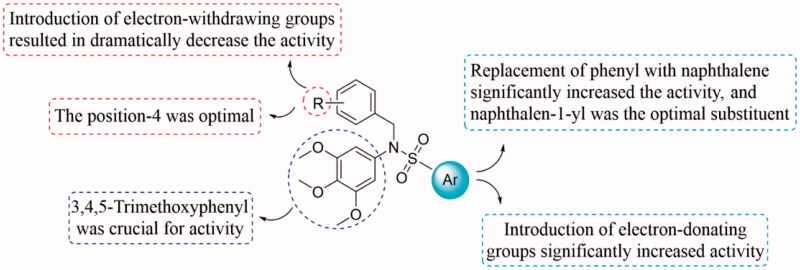
Summarised SARs of this class of compounds.

To explore the effect of the substituents in phenyl ring on the inhibitory activity, various substituents including 3-methoxy, 4-methyl, 3-fluoro, 4-bromo, 4-chloro, 3-bromo, 4-fluoro, 4-cyano, and 2-nitro were introduced into the targeted compounds (**8a**-**8i**). Shifting methoxy group to the 3- position (**8a**) decreased the inhibitory activity. The replacement of the 4-methoxy (**5c**) with 4-methyl group (**8 b**) resulted in a slightly decrease of the inhibitory activity. Introduction of electron-withdrawing groups such as 3-fluoro (**8c**), 4-bromo (**8d**), 4-chloro (**8e**), 3-bromo (**8f**), 4-fluoro (**8 g**), 4-cyano (**8 h**), and 2-nitro (**8i**) into the phenyl ring, resulted in dramatically decrease the inhibitory activity. Compared the inhibitory activity of **5c** (4-methoxy) and **8d** (4-bromo) with **8a** (3-methoxy) and **8f** (3-bromo), the results were shown that the substituent group on the 4-position of the phenyl ring is more beneficial to the antiproliferative activity. The detailed SARs were summarised in Figure 4. The information of SARs provided us a guideline to improve the inhibitory activity in the future structural modification.

In order to verify the safety profile of this class of compounds, the most potent compound **5c** was selected to test its cytotoxicity against human normal liver cell line (LO2). The result was shown that compound **5c** exhibited moderate cytotoxic activity against human normal liver cell (LO2) with IC_50_ value of 12.73 ± 3.26 µM. While, **5c** displayed potent anticancer activity against MCF-7 and A549 cell lines with IC_50_ value of 0.51 ± 0.03 and 0.33 ± 0.01 µM, respectively. Hence, we could conclude that these compounds have good safety for potential application in the treatment of tumour cells.

### 2.3. Inhibition of tubulin polymerisation

**Figure 5.**
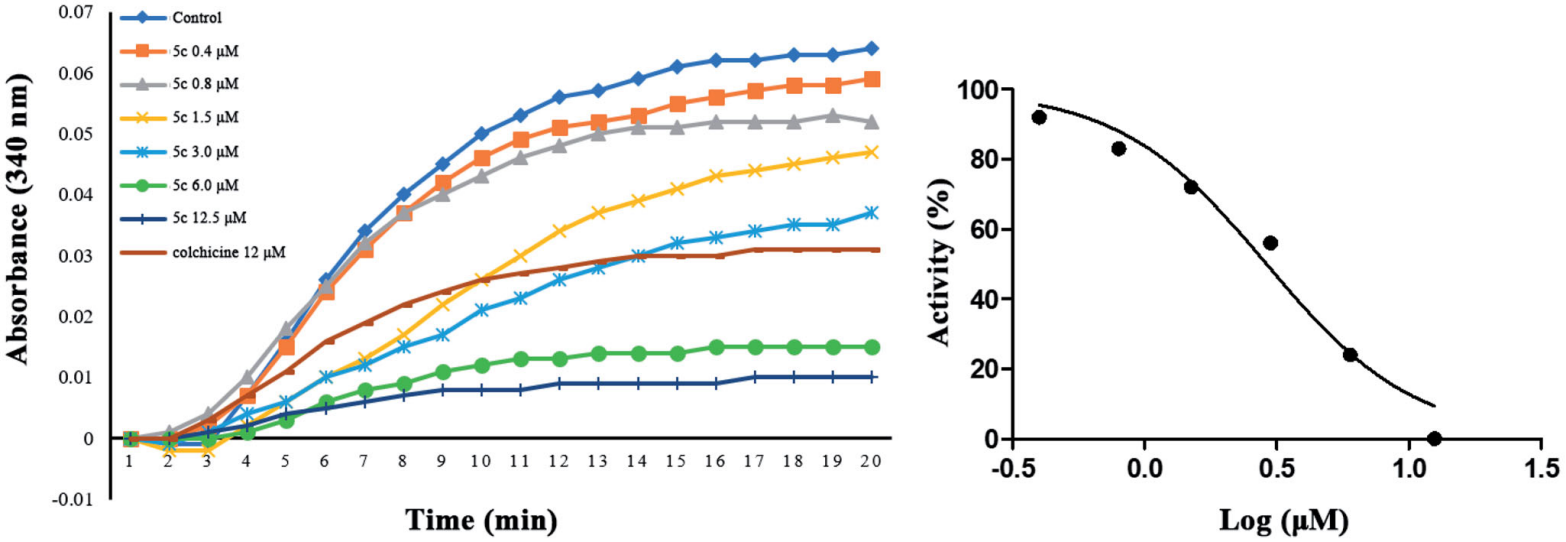
Tubulin polymerisation inhibitory activities of compound **5c** and colchicine.

To examine whether tubulin is the target of this class of compounds, the *in vitro* tubulin polymerisation inhibitory activity of **5c** was evaluated using tubulin polymerisation assay ^28^. Meanwhile, tubulin polymerisation inhibitor colchicine was used as positive control. As shown in Figure 5, compared with the control, the absorbance values at 340 nm of tubulin gradually decreased after incubation with different concentrations of **5c** or colchicine. The results shown that **5c** exhibited a significant ability to inhibit tubulin polymerisation in a concentration-dependent manner with IC_50_ values of 2.8 µM, as compared to colchicine (IC_50_ = 9.3 µM). Besides, **5c** and colchicine have similar effects on inhibit tubulin polymerisation, indicating that **5c** was a microtubulin-destabilizing agent.

### 2.4. Cell cycle arrest

**Figure 6.**
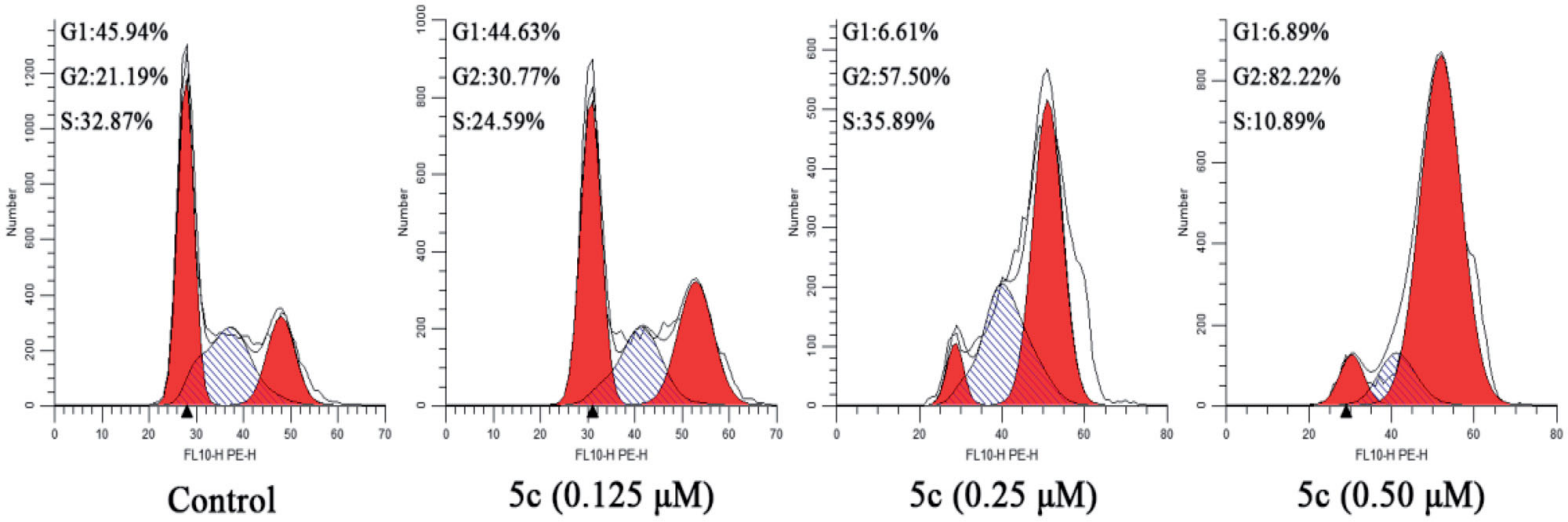
Effect of compound **5c** on cell cycle arrest in MCF-7 cells.

Due to microtubules play an important role in eukaryotic cell division, tubulin polymerisation inhibitors can disrupt regulated cell cycle distribution and block the cell cycle in G2/M phase ^21^^,^^29^. Therefore, the effect of compound **5c** on the cell cycle of MCF-7 cancer cells was evaluated by using flow cytometry. As shown in Figure 6, after treated with different concentrations of compound **5c** (0.125 µM, 0.25 µM or 0.5 µM), the G2/M population in MCF-7 cancer cells increased from 21.19% (control) to 30.77% (0.125 µM), 57.50% (0.25 µM), and 82.22% (0.5 µM), respectively. The results indicate that compound **5c** can arrest cell cycle at G2/M phase in a dose-dependent manner.

### 2.5. Cell apoptosis

**Figure 7.**
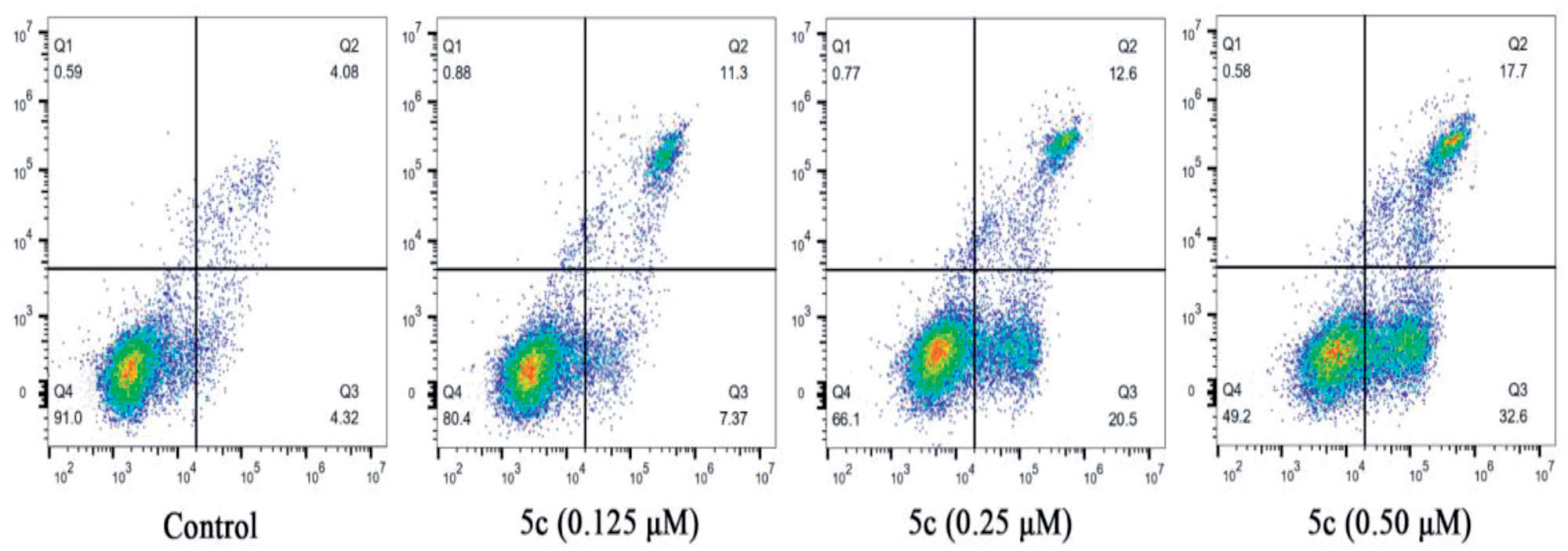
Effect of compound **5c** on cell apoptosis in MCF-7 cells.

Since many literatures have reported that tubulin polymerisation inhibitors are able to induce cellular apoptosis ^22^^,^^30^, the Annexin V-FITC/PI assay was carried out to examine the influence of compound **5c** on cell apoptosis in MCF-7 cancer cells. As shown in Figure 7, when the cells were treatment with compound **5c** in the concentration of 0.125 µM, 0.25 µM, or 0.5 µM, the total numbers of early (the lower right quadrant) and late apoptotic cells (the upper right quadrant) were 18.67%, 33.1% and 50.3%, respectively, whereas that of control was only 8.4%. These results indicated that compound **5c** effectively induced cell apoptosis in MCF-7 cells in a dose-dependent manner.

### 2.6. Molecular docking

**Figure 8.**
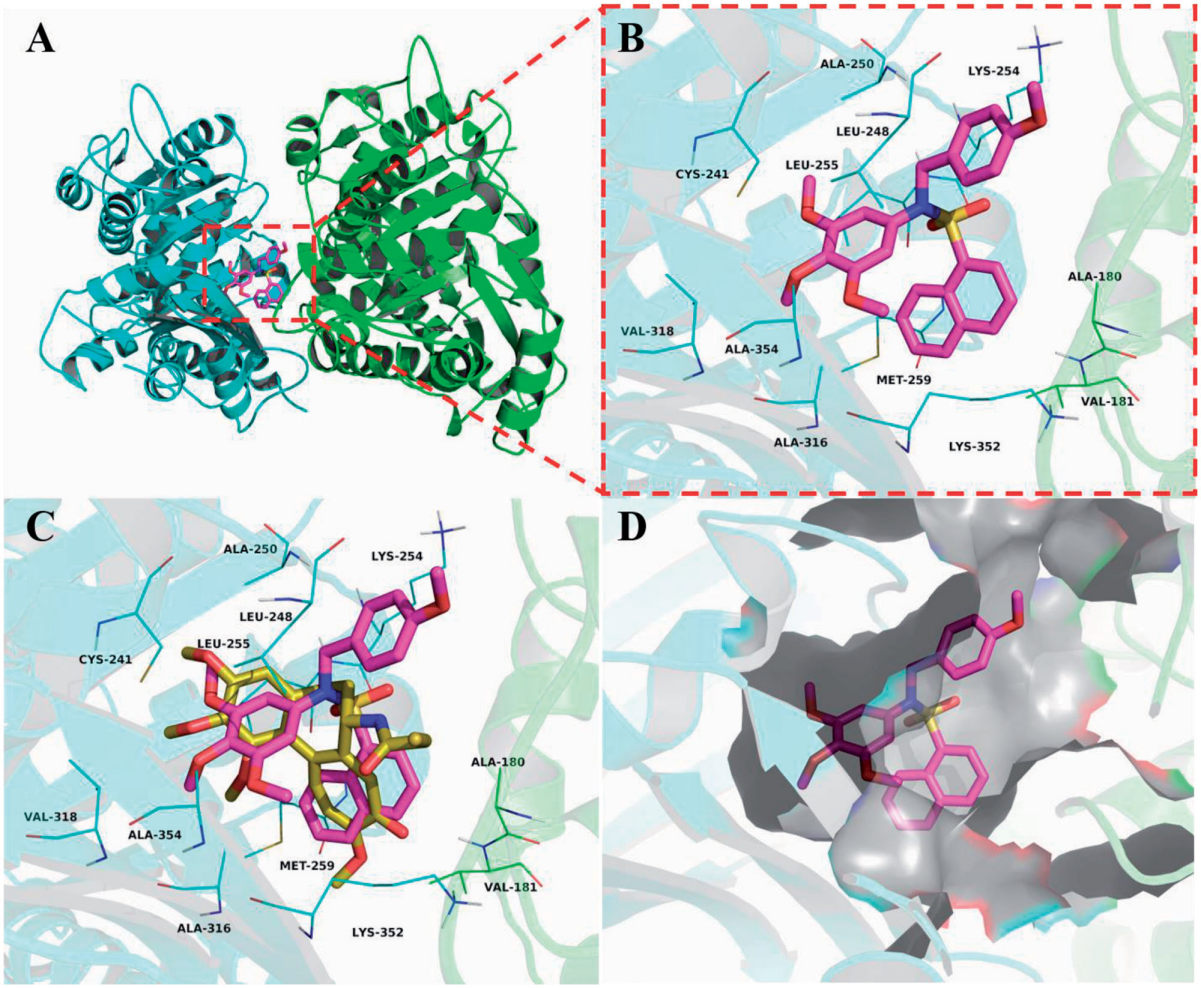
Compound **5c** was docked to the binding pocket of the tubulin (α: green; β: cyan). (A) Overall structure of tubulin with **5c**. (B) Binding pose of **5c** at colchicine binding site. (C) Superimposed pose of **5c** (rose red) and colchicine (yellow-orange) in the binding site. (D) Binding pose of **5c** in the surface of colchicine binding pocket.

To elucidate the binding mode of this class of compounds, molecular docking simulations of compound **5c** with tubulin were performed. Colchicine was first docked into the colchicine binding site of tubulin. The result was shown that co-crystallized conformation of colchicine was reproduced approximately (RMSD: 1.10 Å), indicating that this protocol of molecular docking is credible. Then, the theoretical binding mode between **5c** and tubulin was investigated, and the estimated binding energy was −9.6 kcal·mol^−1^. As shown in Figure 8, Compound **5c** adopted an “Y-shaped” conformation in the colchicine pocket of tubulin. Compound **5c** located at the hydrophobic pocket, surrounded by the residues A/Ala-180, A/Val-181, B/Leu-248, B/Ala-250, B/Leu-255, B/Ala-316, B/Val-318, and B/Ala-354, forming a strong hydrophobic binding. Detailed analysis showed that the phenyl and naphthyl groups of **5c** formed cation-π interactions with the residues Lys-254 and Lys-352, respectively. All these interactions helped **5c** to anchor in the colchicine binding site of tubulin.

### 2.7. Molecular dynamics (MD) simulations

**Figure 9.**
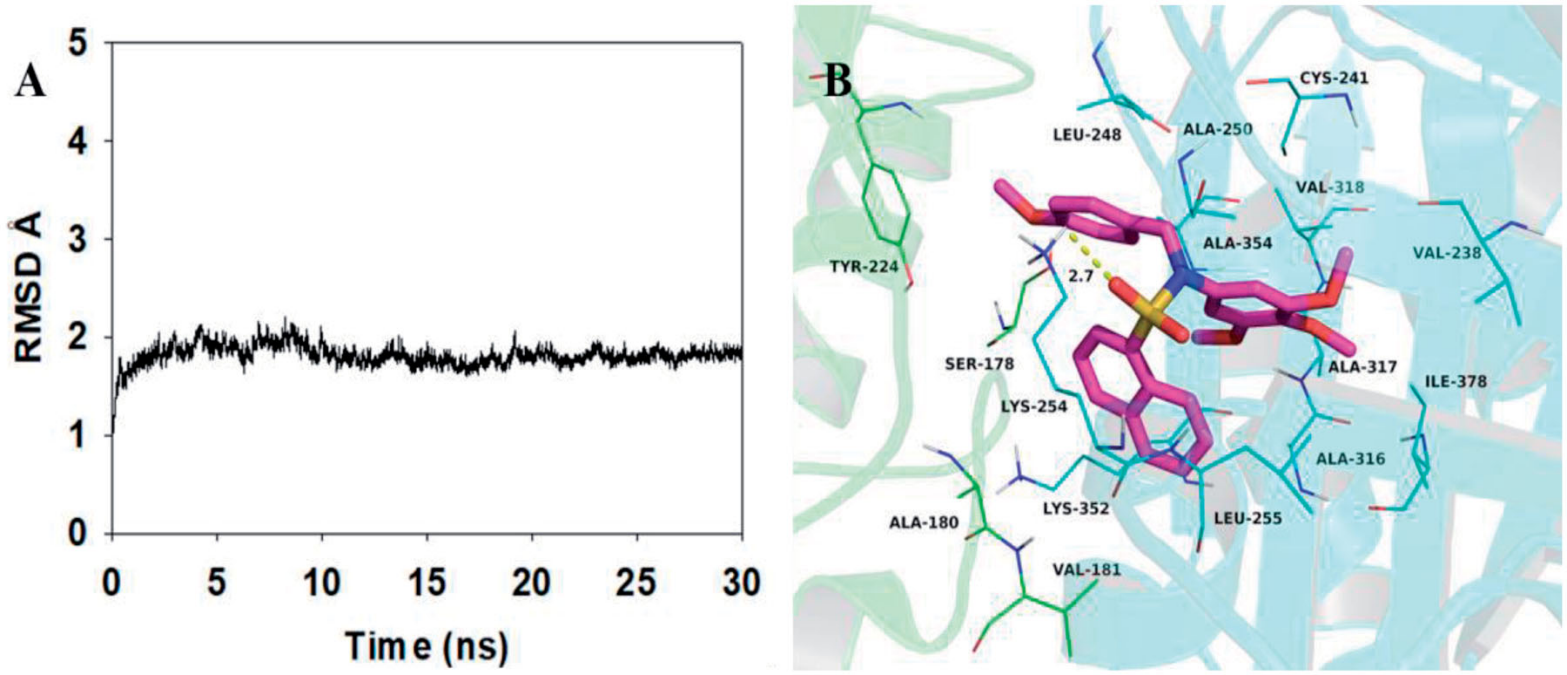
Molecular docking and molecular dynamics refinement of compound **5c** with tubulin. (A) The root-mean-square deviation (RMSD) of all the atoms of tubulin-**5c** complex with respect to its initial structure as function of time. (B) Molecular dynamics results of tubulin-**5c** complex.

To explore the potential binding mode between **5c** and tubulin, molecular docking and molecular dynamics simulations were performed using the AutoDock vina 1.1.2 and Amber12 software package. The preferential binding mechanism of tubulin with **5c** was determined by 30-ns molecular dynamics simulations based on the docking results. To explore the dynamic stability of the models and to ensure the rationality of the sampling strategy, the root-mean-square deviation (RMSD) value of the protein backbone based on the starting structure along the simulation time was calculated and plotted in Figure 9(A). The result was shown that the protein structure of the system was stabilised during the simulation.

The theoretical binding mode between **5c** and tubulin was shown in Figure 9(B). Compound **5c** adopted a compact conformation in the pocket of tubulin. The naphthyl group of **5c** located at the hydrophobic pocket, surrounded by the residues A/Ala-180, A/Val-181, B/Val-238, B/Leu-248, B/Ala-250, B/Leu-255, B/Ala-316, B/Ala-317, B/Val-318 and B/Ala-354, forming a strong hydrophobic binding. Detailed analysis showed that the phenyl group of **5c** formed a cation-π interaction with the residue Lys-254. It was shown that the residue Lys-254 (bond length: 2.7 Å) formed a hydrogen bond with **5c**, which was the main interaction between **5c** and tubulin. All in all, the above molecular dynamics simulation gave us rational explanation of the interaction between **5c** and tubulin, which provided valuable information for further development of tubulin polymerisation inhibitors.

## 3. Conclusion

In summary, a new series of sulphonamide derivatives bearing naphthalene moiety have been synthesised and and characterised by ^1^H NMR, ^13 ^C NMR, HRMS, and elemental analysis. All of the title compounds were screened for antiproliferative activity against human breast cancer cells (MCF-7) and human non-small cell lung carcinoma cells (A549) by using CCK-8 method. Among all synthesised compounds, compound **5c** with naphthalen-1-yl moiety exhibited the most potent antiproliferative activity against MCF-7 and A549 cell line, with IC_50_ values of 0.51 ± 0.03 µM and 0.33 ± 0.01 µM, respectively. SAR studies suggested that the naphthalen-1-yl and 4-methoxybenzyl at the sulphonamide played an important role for the potent antiproliferative activity. Tubulin polymerisation assay revealed that compound was a microtubulin-destabilizing agent with IC_50_ value of 2.8 µM. Further mechanism evaluation demonstrated that **5c** can significantly arrest the cell cycle at G2/M phase and induce apoptosis in MCF-7 cancer cells. Additionally, molecular modelling results showed that **5c** binds well to the colchicine-binding site of tubulin. Hence, these results suggest that **5c** could be used as a promising lead compound for further investigation in anticancer drug development.

## 4. Experimental

### 4.1. 4-Methoxy-*N*-(3,4,5-trimethoxyphenyl)benzamide (3)

To a solution of 3,4,5-trimethoxyaniline **1** (10 mmol) and Et_3_N (20 mmol) in THF (50 ml) were added 4-methoxybenzoyl chloride **2** (10 mmol) and the reacting mixture was stirred at room temperature for 4 h. Then, the solvent was removed under reduced pressure, and water was added to the reaction mixture and extracted 3 times with ethyl acetate. The combined organic layers were dried, filtered, and concentrated. The residue was purified by chromatography on silica gel with EtOAc/petroleum ether = 1:1 to give compound **3** as white solid (yield 88%).

### 4.2. 3,4,5-Trimethoxy-*N*-(4-methoxybenzyl)aniline (4)

A mixture of LiAlH_4_ (2 mmol) in anhydrous THF (10 ml) was stirred in an ice bath for 10 min. Then, a solution of compound **3** (1 mmol) in 10 ml of THF was added dropwise to the mixture. After completion of dropwise addition, the mixture was stirred at 0 °C for 30 min then refluxed at 70–80 °C for 2 h. After completion of the reaction, the solvent was evaporated and the organic product was extracted with ethyl acetate. The combined organic layers were dried over sodium sulphate and evaporated to afford black oil, which was used directly for the next step without further purification.

### 4.3. General procedure for the synthesis of 5a-5e

To a solution of compound **4** (2 mmol), DMAP (0.2 mmol), and Et_3_N (2 mmol) in THF (50 ml) was added commercially available aryl sulphonyl chloride (2 mmol) and the reacting mixture was stirred at room temperature for 12 h. The solvent was evaporated and water was added to the reaction mixture. The organic material was extracted with ethyl acetate, and the organic layer was dried over Na_2_SO_4_ and concentrated under vacuum. The residue was purified by chromatography to give the title product **5a**-**5e**.

### 4.4. *N*-(3,4,5-Trimethoxyphenyl)naphthalene-1-sulphonamide (7)

To a solution of 3,4,5-trimethoxyaniline **1** (10 mmol) and Et_3_N (10 mmol) in CH_2_Cl_2_ (100 ml) was added naphthalene-1-sulphonyl chloride **6** (10 mmol) and the mixture was stirred at room temperature for 6 h. Then, the solution was diluted with CH_2_Cl_2_ and washed with water. After the concentration of the solution, the residue was purified by silica gel column chromatography to give compound 7 as white solid (yield = 43%).

### 4.5. General procedure for the synthesis of 8a-8i

A mixture of **7** (1.0 mmol), K_2_CO_3_ (2.0 mmol), KI (1.0 mmol), and commercially available benzyl halide (1.0 mmol) in acetone (10 ml) was stirred at reflux for 5 h. After completion of the reaction, the mixture was concentrated under reduced pressure and the residue was purified by chromatography to give the title compounds **8a**-**8i**.

## Supplementary Material

Supplemental Material
