# Supplementary material for: Synthesis, biological evaluation and molecular docking investigation of new sulphonamide derivatives bearing naphthalene moiety as potent tubulin polymerisation inhibitors
Source: J Enzyme Inhib Med Chem. 2021 Jun 22;36(1):1402–10. doi: 10.1080/14756366.2021.1943378 (PMC8231400; doi:10.1080/14756366.2021.1943378)
Supplement: Supplemental Material [file IENZ_A_1943378_SM6566.pdf]

Supplemental Material

1. Experimental section.

1.1. Chemistry.

All starting materials and reagents were purchased from commercial suppliers. Nuclear magnetic resonance spectra (NMR) were recorded on a JNM spectrometer (400 MHz) with TMS as an external reference and reported in parts per million. High-resolution mass spectra (HRMS) were recorded on Bruker MicroQTOF II and Shimadzu LCMS-IT-TOF using ESI method.

N-(4-Methoxybenzyl)-4-methyl-*N*-(3,4,5-trimethoxyphenyl)benzenesulfonamide (**5a**)

Solid, mp 172-173 ^{o}C. ^{1}H NMR (d_6-DMSO, 400 MHz) δ: 2.37 (s, 3H), 3.51 (s, 6H), 3.55 (s, 3H), 3.64 (s, 3H), 4.60 (s, 2H), 6.17 (s, 2H), 6.76 (d, 2H, J = 7.2 Hz), 7.12 (d, 2H, J = 7.2 Hz), 7.39 (d, 2H, J = 7.2 Hz), 7.53 (d, 2H, J = 7.2 Hz); ^{13}C NMR (d_6-DMSO, 100 MHz) δ: 21.56, 53.73, 55.50, 56.39, 60.53, 107.00, 114.20, 128.14, 128.65, 130.12, 130.20, 134.85, 135.60, 137.41, 144.11, 152.87, 159.08; HRMS (ESI) calcd for $[M+H]^+$ $C_{24}H_{28}NO_6S^+$: 458.1632 found 458.1636. Elemental analysis for $C_{24}H_{27}NO_6S$: C, 63.00; H, 5.95; N, 3.06; S, 7.01 found C, 62.46; H, 5.585; N, 3.04; S, 6.637.

N-(4-Methoxybenzyl)-*N*-(3,4,5-trimethoxyphenyl)benzenesulfonamide (**5b**)

Solid, mp 181-182 ^{o}C. ^{1}H NMR (d_6-DMSO, 400 MHz) δ: 3.51 (s, 6H), 3.56 (s, 3H), 3.65 (s, 3H), 4.64 (s, 2H), 6.16 (s, 2H), 6.77 (d, 2H, J = 7.2 Hz), 7.12 (d, 2H, J = 7.2 Hz), 7.59-7.61 (m, 2H), 7.67-7.68 (m, 3H); ^{13}C NMR (d_6-DMSO, 100 MHz) δ: 53.89, 55.51, 56.36, 60.53, 106.99, 114.21, 128.07, 128.59, 129.81, 130.15, 133.71, 134.70, 137.43, 138.41, 152.89, 159.10; HRMS (ESI) calcd for $[M+H]^+$ $C_{23}H_{26}NO_6S^+$: 444.1475

found 444.1434. Elemental analysis for $C_{23}H_{25}NO_6S$: C, 62.29; H, 5.68; N, 3.16; S, 7.23 found C, 61.24; H, 5.338; N, 3.03; S, 7.035.

N-(4-Methoxybenzyl)-*N*-(3,4,5-trimethoxyphenyl)naphthalene-1-sulfonamide (**5c**)

Solid, mp 157-160 °C. ^{1}H NMR (d_6-DMSO, 400 MHz) δ: 3.33 (s, 6H), 3.53 (s, 3H), 3.63 (s, 3H), 4.69 (s, 2H), 6.11 (s, 2H), 6.75 (d, 2H, J = 8.4 Hz), 7.09 (d, 2H, J = 8.4 Hz), 7.48 (td, 1H, J = 8.0 Hz, 1.2 Hz), 7.60 (td, 1H J = 8.0 Hz, 1.2 Hz), 7.65 (t, 1H, J = 8.0 Hz), 8.05 (d, 1H, J = 8.0 Hz), 8.14 (d, 1H, J = 8.0 Hz), 8.16 (dd, 1H, J = 8.0 Hz, 1.2 Hz), 8.26 (d, 1H, J = 8.0 Hz); ^{13}C NMR (d_6-DMSO, 100 MHz) δ: 53.37, 55.50, 56.20, 60.61, 107.41, 114.18, 125.24, 125.36, 127.40, 128.15, 128.59, 128.83, 129.35, 130.24, 131.17, 133.95, 134.08, 134.32, 135.17, 137.59, 152.80, 159.10; HRMS (ESI) calcd for $[M+Na]^+$ $C_{27}H_{27}NNaO_6S^+$: 516.1451 found 516.1432. Elemental analysis for $C_{27}H_{27}NO_6S$: C, 65.70; H, 5.51; N, 2.84; S, 6.50 found C, 66.04; H, 5.429; N, 2.71; S, 6.335.

N-(4-Methoxybenzyl)-*N*-(3,4,5-trimethoxyphenyl)naphthalene-2-sulfonamide (**5d**)

Solid, mp 188-189 °C. ^{1}H NMR (d_6-DMSO, 400 MHz) δ: 3.42 (s, 6H), 3.54 (s, 3H), 3.64 (s, 3H), 4.67 (s, 2H), 6.18 (s, 2H), 6.76 (d, 2H, J = 8.0 Hz), 7.13 (d, 2H, J = 8.0 Hz), 7.64-7.70 (m, 3H), 8.04 (d, 1H, J = 8.0 Hz), 8.14 (t, 2H, J = 8.0 Hz), 8.37 (s, 1H); ^{13}C NMR (d_6-DMSO, 100 MHz) δ: 53.90, 55.50, 56.31, 60.53, 107.15, 114.21, 123.52, 128.20, 128.37, 128.60, 129.37, 129.58, 129.73, 129.87, 130.17, 132.31, 134.80, 134.98, 135.54, 137.51, 152.90, 159.10; HRMS (ESI) calcd for $[M+Na]^+$ $C_{27}H_{27}NNaO_6S^+$: 516.1451 found 516.1434. Elemental analysis for $C_{27}H_{27}NO_6S$: C, 65.70; H, 5.51; N, 2.84; S, 6.50 found C, 64.95; H, 5.202; N, 2.73; S, 6.426.

4-Methoxy-*N*-(4-methoxybenzyl)-*N*-(3,4,5-trimethoxyphenyl)benzenesulf

onamide (**5e**)

Solid, mp 179-182 ^{o}C. ^{1}H NMR (d_6-DMSO, 400 MHz) δ: 3.53 (s, 6H), 3.55 (s, 3H), 3.64 (s, 3H), 3.82 (s, 3H), 4.58 (s, 2H), 6.18 (s, 2H), 6.76 (d, 2H, J = 8.8 Hz), 7.10 (d, 2H, J = 8.8 Hz), 7.12 (d, 2H, J = 8.8 Hz), 7.58 (d, 2H, J = 8.8 Hz); ^{13}C NMR (d_6-DMSO, 100 MHz) δ: 53.64, 55.50, 56.30, 56.40, 60.53, 106.99, 114.18, 114.90, 128.68, 130.11, 130.34, 135.01, 137.36, 152.87, 159.05, 163.29; HRMS (ESI) calcd for [M+Na]$^+$ $C_{24}H_{27}NNaO_7S^+$: 496.1400 found 496.1390. Elemental analysis for $C_{24}H_{27}NO_7S$: C, 60.87; H, 5.75; N, 2.96; S, 6.77 found C, 61.39; H, 5.585; N, 2.80; S, 6.496.

N-(3-Methoxybenzyl)-*N*-(3,4,5-trimethoxyphenyl)naphthalene-1-sulfonamide (**8a**)

Solid, mp 154-157 ^{o}C. ^{1}H NMR (d_6-DMSO, 400 MHz) δ: 3.41 (s, 6H), 3.58 (s, 3H), 3.62 (s, 3H), 4.79 (s, 2H), 6.19 (s, 2H), 6.75-6.76 (m, 2H), 6.80 (d, 1H, J = 8.0 Hz), 7.15 (t, 1H, J = 8.0 Hz), 7.56 (t, 1H, J = 8.0 Hz), 7.62-7.68 (m, 2H), 8.08 (d, 1H, J = 8.0 Hz), 8.19 (d, 1H, J = 8.0 Hz), 8.27 (d, 1H, J = 8.0 Hz), 8.31 (d, 1H, J = 8.0 Hz); ^{13}C NMR (d_6-DMSO, 100 MHz) δ: 53.76, 55.40, 56.20, 60.60, 107.28, 113.55, 114.07, 120.96, 125.27, 125.33, 127.43, 128.21, 128.81, 129.39, 129.89, 131.32, 133.75, 134.18, 134.33, 135.27, 137.58, 138.45, 152.83, 159.62; HRMS (ESI) calcd for [M+Na]$^+$ $C_{27}H_{27}NNaO_6S^+$: 516.1451 found 516.1440. Elemental analysis for $C_{27}H_{27}NO_6S$: C, 65.70; H, 5.51; N, 2.84; S, 6.50 found C, 66.16; H, 5.41; N, 2.67; S, 6.404.

N-(4-Methylbenzyl)-*N*-(3,4,5-trimethoxyphenyl)naphthalene-1-sulfonamide (**8b**)

Solid, mp 134-136 ^{o}C. ^{1}H NMR (d_6-DMSO, 400 MHz) δ: 2.21 (s, 3H), 3.37 (s, 6H), 3.57 (s, 3H), 4.76 (s, 2H), 6.17 (s, 2H), 7.04-7.13 (m, 4H), 7.50-7.54 (m, 1H), 7.62-7.69 (m, 3H), 8.09-8.32 (m, 4H); ^{13}C NMR

(d_6-DMSO, 100 MHz) δ: 21.16, 53.67, 56.21, 60.61, 107.37, 125.24, 125.33, 127.41, 128.15, 128.80, 129.36, 131.18, 133.77, 133.88, 134.13, 135.19, 137.14, 137.61, 152.81; HRMS (ESI) calcd for $[M+H]^+$ $C_{27}H_{28}NO_5S^+$: 478.1683 found 478.1647. Elemental analysis for $C_{27}H_{27}NO_5S$: C, 67.90; H, 5.70; N, 2.93; S, 6.71 found C, 67.18; H, 5.362; N, 2.83; S, 6.669.

N-(3-Fluorobenzyl)-*N*-(3,4,5-trimethoxyphenyl)naphthalene-1-sulfonamide (**8c**)

Solid, mp 149-152 °C. ^{1}H NMR (d_6-DMSO, 400 MHz) δ: 3.39 (s, 6H), 3.57 (s, 3H), 4.84 (s, 2H), 6.21 (s, 2H), 7.03-7.08 (m, 2H), 7.12 (d, 1H, *J* = 8.0 Hz), 7.29-7.35 (m, 1H), 7.50-7.54 (m, 1H), 7.65 (t, 1H, *J* = 7.2 Hz), 7.71 (t, 1H, *J* = 8.0 Hz), 8.10 (d, 1H, *J* = 8.0 Hz), 8.15 (d, 1H, *J* = 8.8 Hz), 8.23 (dd, 1H, *J* = 7.6 Hz, 1.2 Hz), 8.32 (d, 1H, *J* = 8.4 Hz); ^{13}C NMR (d_6-DMSO, 100 MHz) δ: 53.38, 56.25, 60.63, 107.31, 114.70, 115.29, 124.80, 125.27, 127.45, 128.21, 128.81, 129.38, 130.77, 131.32, 133.65, 134.09, 134.32, 135.33, 137.74, 139.93, 152.90; HRMS (ESI) calcd for $[M+H]^+$ $C_{26}H_{25}FNO_5S^+$: 482.1432 found 482.1399. Elemental analysis for $C_{26}H_{24}FNO_5S$: C, 64.85; H, 5.02; N, 2.91; S, 6.66 found C, 64.53; H, 4.881; N, 2.80; S, 6.790.

N-(4-Bromobenzyl)-*N*-(3,4,5-trimethoxyphenyl)naphthalene-1-sulfonamide (**8d**)

Solid, mp 126-129 °C. ^{1}H NMR (d_6-DMSO, 400 MHz) δ: 3.34 (s, 6H), 3.53 (s, 3H), 4.75 (s, 2H), 6.14 (s, 2H), 7.17 (d, 2H, *J* = 8.8 Hz), 7.41 (d, 2H, *J* = 8.8 Hz), 7.47 (t, 1H, *J* = 8.0 Hz), 7.58 (t, 1H, *J* = 8.0 Hz), 7.65 (t, 1H, *J* = 8.0 Hz), 8.04 (d, 1H, *J* = 8.4 Hz), 8.12 (d, 1H, *J* = 8.4 Hz), 8.17 (dd, 1H, *J* = 7.2 Hz, 1.2 Hz), 8.25 (d, 1H, *J* = 8.4 Hz); ^{13}C NMR (d_6-DMSO, 100 MHz) δ: 53.24, 56.24, 60.63, 107.33, 121.14, 125.27, 127.42, 128.17, 128.82, 129.36, 131.00, 131.25, 131.75, 133.68, 134.02,

134.31, 135.29, 136.46, 137.72, 152.88; HRMS (ESI) calcd for $[M+H]^+$ $C_{26}H_{25}BrNO_5S^+$: 542.0631 found 542.0597. Elemental analysis for $C_{26}H_{24}BrNO_5S$: C, 57.57; H, 4.46; N, 2.58; S, 5.91 found C, 58.32; H, 4.547; N, 2.44; S, 5.749.

N-(4-Chlorobenzyl)-*N*-(3,4,5-trimethoxyphenyl)naphthalene-1-sulfonamide (**8e**)

Solid, mp 153-154 ^{o}C. ^{1}H NMR (d_6-DMSO, 400 MHz) δ: 3.33 (s, 6H), 3.52 (s, 3H), 4.76 (s, 2H), 6.13 (s, 2H), 7.22 (d, 2H, J = 8.8 Hz), 7.26 (d, 2H, J = 8.8 Hz), 7.47 (t, 1H, J = 8.4 Hz), 7.59 (t, 1H, J = 8.0 Hz), 7.64 (t, 1H, J = 8.0 Hz), 8.04 (d, 1H, J = 8.4 Hz), 8.11 (d, 1H, J = 8.4 Hz), 8.16 (dd, 1H, J = 8.4 Hz, 1.2 Hz), 8.24 (d, 1H, J = 8.4 Hz); ^{13}C NMR (d_6-DMSO, 100 MHz) δ: 53.23, 56.23, 60.64, 107.32, 125.25, 127.45, 128.19, 128.82, 129.36, 130.64, 131.25, 132.60, 134.01, 134.28, 135.30, 135.98, 137.71, 152.87; HRMS (ESI) calcd for $[M+H]^+$ $C_{26}H_{25}ClNO_5S^+$: 498.1136 found 498.1101. Elemental analysis for $C_{26}H_{24}ClNO_5S$: C, 62.71; H, 4.86; N, 2.81; S, 6.44 found C, 62.92; H, 4.733; N, 2.71; S, 6.402.

N-(3-Bromobenzyl)-*N*-(3,4,5-trimethoxyphenyl)naphthalene-1-sulfonamide (**8f**)

Solid, mp 152-155 ^{o}C. ^{1}H NMR (d_6-DMSO, 400 MHz) δ: 3.35 (s, 6H), 3.53 (s, 3H), 4.78 (s, 2H), 6.15 (s, 2H), 7.17-7.24 (m, 2H), 7.36-7.37 (m, 2H), 7.49 (t, 1H, J = 8.4 Hz), 7.60 (t, 1H, J = 8.0 Hz), 7.65 (t, 1H, J = 8.0 Hz), 8.06 (d, 1H, J = 8.0 Hz), 8.15-8.19 (m, 2H), 8.26 (d, 1H, J = 8.0 Hz); ^{13}C NMR (d_6-DMSO, 100 MHz) δ: 53.23, 56.21, 60.63, 107.31, 121.96, 125.26, 127.48, 127.80, 128.23, 128.76, 129.41, 130.84, 131.05, 131.36, 131.45, 133.58, 134.02, 134.31, 135.38, 137.68, 139.79, 152.89; HRMS (ESI) calcd for $[M+H]^+$ $C_{26}H_{25}BrNO_5S^+$: 542.0631 found 542.0604.

N-(4-Fluorobenzyl)-*N*-(3,4,5-trimethoxyphenyl)naphthalene-1-sulfonami

de (**8g**)

Solid, mp 145-148 ^{o}C. ^{1}H NMR (d_6-DMSO, 400 MHz) δ: 3.34 (s, 6H), 3.53 (s, 3H), 4.76 (s, 2H), 6.13 (s, 2H), 7.04 (t, 2H, J = 8.8 Hz), 7.23 (d, 1H, J = 8.4 Hz), 7.24 (d, 1H, J = 8.4 Hz), 7.49 (t, 1H, J = 8.4 Hz), 7.60 (t, 1H, J = 7.2 Hz), 7.65 (t, 1H, J = 8.0 Hz), 8.05 (d, 1H, J = 8.4 Hz), 8.17 (t, 2H, J = 8.8 Hz), 8.26 (d, 1H, J = 8.0 Hz); ^{13}C NMR (d_6-DMSO, 100 MHz) δ: 53.15, 56.22, 60.62, 107.40, 115.52, 115.73, 125.25, 125.33, 127.42, 128.18, 128.83, 129.36, 130.86, 130.94, 131.23, 133.10, 133.81, 134.01, 134.32, 135.25, 137.68, 152.86; HRMS (ESI) calcd for $[M+Na]^+$ $C_{26}H_{24}FNNaO_5S^+$: 504.1251 found 504.1243. Elemental analysis for $C_{26}H_{24}FNO_5S$: C, 64.85; H, 5.02; N, 2.91; S, 6.66 found C, 59.42; H, 4.479; N, 2.59; S, 6.285.

N-(4-Cyanobenzyl)-*N*-(3,4,5-trimethoxyphenyl)naphthalene-1-sulfonamide (**8h**)

Solid, mp 156-159 ^{o}C. ^{1}H NMR (d_6-DMSO, 400 MHz) δ: 3.35 (s, 6H), 3.52 (s, 3H), 4.88 (s, 2H), 6.18 (s, 2H), 7.44-7.48 (m, 3H), 7.60 (t, 1H, J = 8.0 Hz), 7.64 (t, 1H, J = 8.0 Hz), 7.70 (d, 2H, J = 8.4 Hz), 8.05 (d, 1H, J = 8.4 Hz), 8.09 (d, 1H, J = 8.4 Hz), 8.18 (d, 1H, J = 8.0 Hz), 8.27 (d, 1H, J = 8.0 Hz); ^{13}C NMR (d_6-DMSO, 100 MHz) δ: 53.51, 56.26, 60.62, 107.23, 110.74, 119.20, 125.26, 127.45, 128.20, 128.83, 129.37, 129.54, 131.34, 132.82, 133.50, 134.08, 134.31, 135.38, 137.75, 142.98, 152.93; HRMS (ESI) calcd for $[M+Na]^+$ $C_{27}H_{24}N_2NaO_5S^+$: 511.1298 found 511.1272. Elemental analysis for $C_{27}H_{24}N_2O_5S$: C, 66.38; H, 4.95; N, 5.73; S, 6.56 found C, 66.61; H, 4.921; N, 5.45; S, 6.363.

N-(2-Nitrobenzyl)-*N*-(3,4,5-trimethoxyphenyl)naphthalene-1-sulfonamide (**8i**)

Solid, mp 125-128 ^{o}C. ^{1}H NMR (d_6-DMSO, 400 MHz) δ: 3.33 (s, 6H), 3.51 (s, 3H), 5.12 (s, 2H), 6.17 (s, 2H), 7.43-7.46 (m, 2H), 7.59 (t, 1H, J

= 8.0 Hz), 7.62 (t, 1H, *J* = 8.0 Hz), 7.67 (t, 1H, *J* = 8.0 Hz), 7.72 (d, 1H, *J* = 7.6 Hz), 7.84 (d, 1H, *J* = 7.6 Hz), 8.06 (t, 2H, *J* = 8.0 Hz), 8.13 (d, 1H, *J* = 7.6 Hz), 8.27 (d, 1H, *J* = 8.0 Hz); ^{13}C NMR (d_6-DMSO, 100 MHz) δ: 51.01, 56.22, 60.63, 107.33, 125.00, 125.21, 125.24, 127.40, 128.17, 129.01, 129.31, 129.51, 131.34, 131.41, 131.59, 133.09, 133.89, 134.12, 134.28, 135.48, 137.89, 149.07, 152.87; HRMS (ESI) calcd for $[M+Na]^+$ $C_{26}H_{24}N_2NaO_7S^+$: 531.1196 found 531.1162. Elemental analysis for $C_{26}H_{24}N_2O_7S$: C, 61.41; H, 4.76; N, 5.51; S, 6.30 found C, 61.52; H, 4.702; N, 5.21; S, 6.100.

1.2. In vitro antiproliferative assay

Human breast cancer cell line (MCF-7) and human non-small cell lung carcinoma cell line (A549) were used to determine the antiproliferative of the test compounds by CCK-8 assay. The cells were cultured in RPMI media with 10% fetal bovine serum at 37 °C and 5% CO2 in a humidify environment. In a word, cells were seeded in 96-well plates (1×10^5 cells/well) and cultured for overnight. After incubation, the cells were treated with test compounds and positive control for 48 h. Finally, cell viability was determined by the CCK-8 method.

1.3. *In vitro* tubulin polymerization inhibitory assay

Tubulin protein was mixed with different concentrations of compound **5c** or colchicine in PEM buffer (100 mM PIPES, 1 mM $MgCl_2$, and 1 mM EGTA) with 1 mM GTP and 5% glycerol. Then the plate was immediately transferred to the spectrophotometer and microtubule polymerization was monitored under 340 nm at 37 °C every 1 min over 20 min.

1.4. Cell cycle assay

MCF-7 cells were seeded into 6-well plates and incubated for 24 h, and then the cells were treated with different concentrations of compound **5c**

for 24 h. After incubation, the cells were harvested by centrifugation, and fixed in ice-cold 70% ethanol overnight. After removed the ethanol, the cells were incubated with RNase and propidium iodide. Finally, the samples were analyzed by flow cytometry.

1.5. Cell apoptosis assay

MCF-7 cells were seeded into 6-well plates and incubated for 24 h, and then the cells were treated with different concentrations of compound **5c** for 24 h. Subsequently, the cells were incubated with Annexin-V/FITC binding buffer and PI staining solution at room temperature. Finally, the stained cells were analyzed using flow cytometry.

1.6. Molecular docking

Molecular docking studies were performed to investigate the binding mode between the compound and tubulin using Autodock vina 1.1.2. The three-dimensional (3D) coordinate of tubulin (PDB ID: 1SA0) was downloaded from Protein Data Bank (www.rcsb.org). The 3D structure of the compounds was obtained by ChemBioDraw Ultra 14.0 and ChemBio3D Ultra 14.0 softwares. The AutoDockTools 1.5.6 package was employed to generate the docking input files. The search grid of tubulin was identified as center_x: 118.921, center_y: 89.718, and center_z: 5.932 with dimensions size_x: 15, size_y: 15, and size_z: 15. The value of exhaustiveness was set to 20. For Vina docking, the default parameters were used if it was not mentioned. The best-scoring pose as judged by the Vina docking score was chosen and visually analyzed using PyMoL 1.7.6 software (www.pymol.org).

1.7 Molecular dynamics

The Amber 12 [1-3] and AmberTools 13 programs were used for MD simulations of the selected docked pose. MBPPS was first prepared by ACPYPE [4], a tool based on ANTECHAMBER [5, 6] for generating

automatic topologies and parameters in different formats for different molecular mechanics programs, including calculation of partial charges. Then, the forcefield “leaprc.gaff” (generalized amber forcefield) was used to prepare the ligand, while “leaprc.ff12SB” was used for the receptor. The system was placed in a rectangular box (with a 10.0 Å boundry) of TIP3P water using the “SolvateOct” command with the minimum distance between any solute atoms. Equilibration of the solvated complex was done by carrying out a short minimization (500 steps of each steepest descent and conjugate gradient method), 500 ps of heating, and 50 ps of density equilibration with weak restraints using the GPU (NVIDIA® Tesla K20c) accelerated PMEMD (Particle Mesh Ewald Molecular Dynamics) module. At last, 20 ns of MD simulations were carried out.

Table S1. ^{1}H and ^{13}C NMR spectral data of compound **5c** (400 and 100 MHz in DMSO-*d6*)

**Position**	δ_C (ppm)	δ_H (ppm)
1	133.95	
2	131.17	8.16 dd (8.0 Hz, 1.2 Hz)
3	125.24	7.65 t (8.0 Hz)
4	135.17	8.26 d (8.0 Hz)
5	129.35	8.05 d (8.0 Hz)
6	127.40	7.60 td (8.0 Hz, 1.2 Hz)
7	128.15	7.48 td (8.0 Hz, 1.2 Hz)
8	125.36	8.14 d (8.0 Hz)
9	128.83	
10	134.32	
11	53.37	4.69 s
1′	128.59	

2′	130.24	7.09 d (8.4 Hz)
3′	114.18	6.75 d (8.4 Hz)
4′	159.10	
5′	114.18	6.75 d (8.4 Hz)
6′	130.24	7.09 d (8.4 Hz)
1″	134.08	
2″	107.41	6.11 s
3″	152.80	
4″	137.59	
5″	152.80	
6″	107.41	6.11 s
OCH_3-C4′	55.50	3.63 s
OCH_3-C4″	60.61	3.53 s
OCH_3-C3″	56.20	3.33 s
OCH_3-C5″	56.20	3.33 s

Table S2. HMBC correlations (H→C) of compound **5c**.

**Position**	HMBC (H→C)	**Position**	HMBC (H→C)
1		4′	
2	C (4)	5′	C (1′, 4′, 2′, 6′)
3	C (1, 10)	6′	C (1′, 4′)
4	C (2, 9)	1″	
5	C (4, 6, 9)	2″	C (1″, 4″, 2″, 6″, 3″, 5″)
6	C (8)	3″	
7	C (9)	4″	
8	C (7)	5″	
9		6″	C (1″, 4″, 2″, 6″, 3″, 5″)
10		OCH_3-C4′	C (4′)

11	C (2′, 6′, 1″)	OCH_3-C4″	C (3″, 4″)
1′		OCH_3-C3″	C (3″)
2′	C (1′, 4′)	OCH_3-C5″	C (5″)
3′	C (1′, 4′, 2′, 6′)		

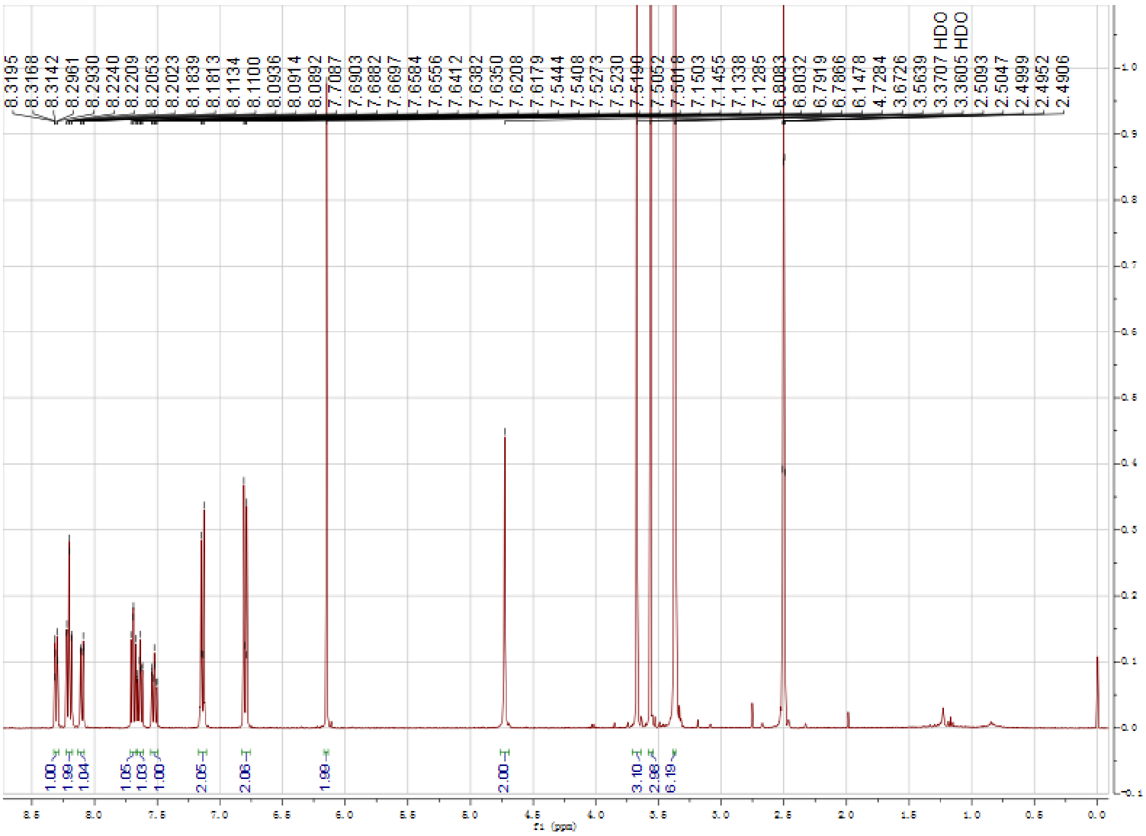

Figure S1. ^{1}H NMR of compound **5c**

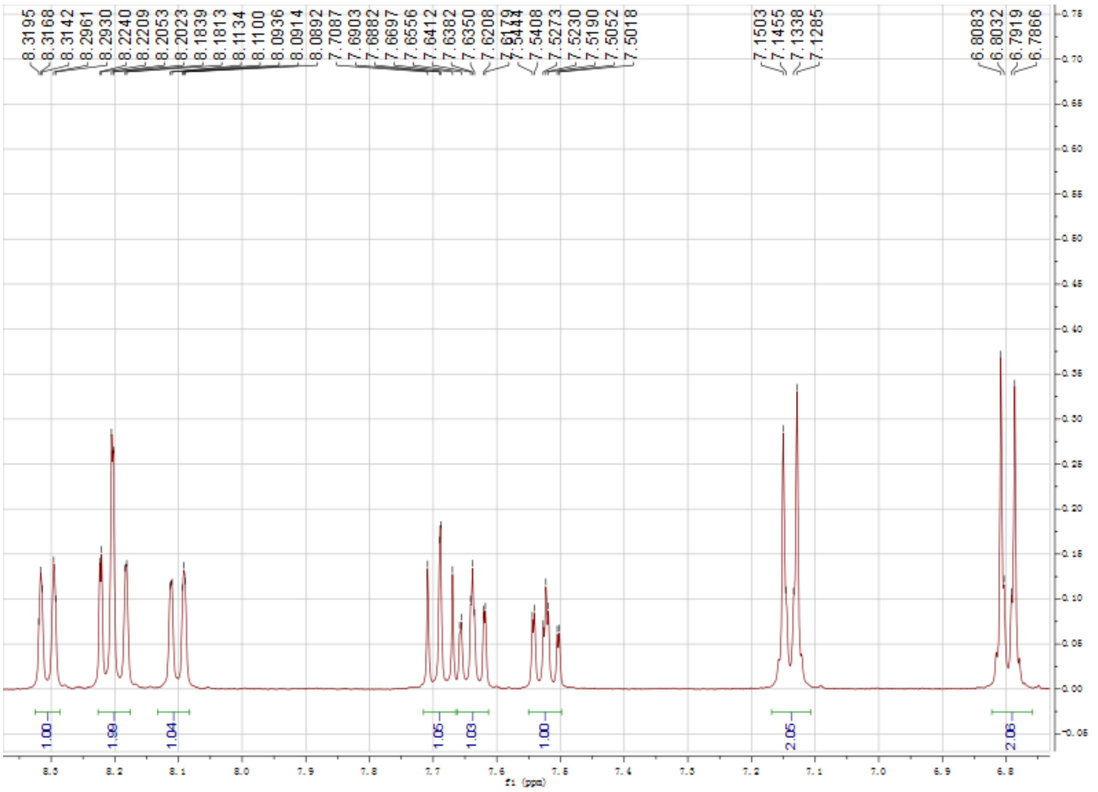

Figure S2. ^{1}H NMR of compound **5c**

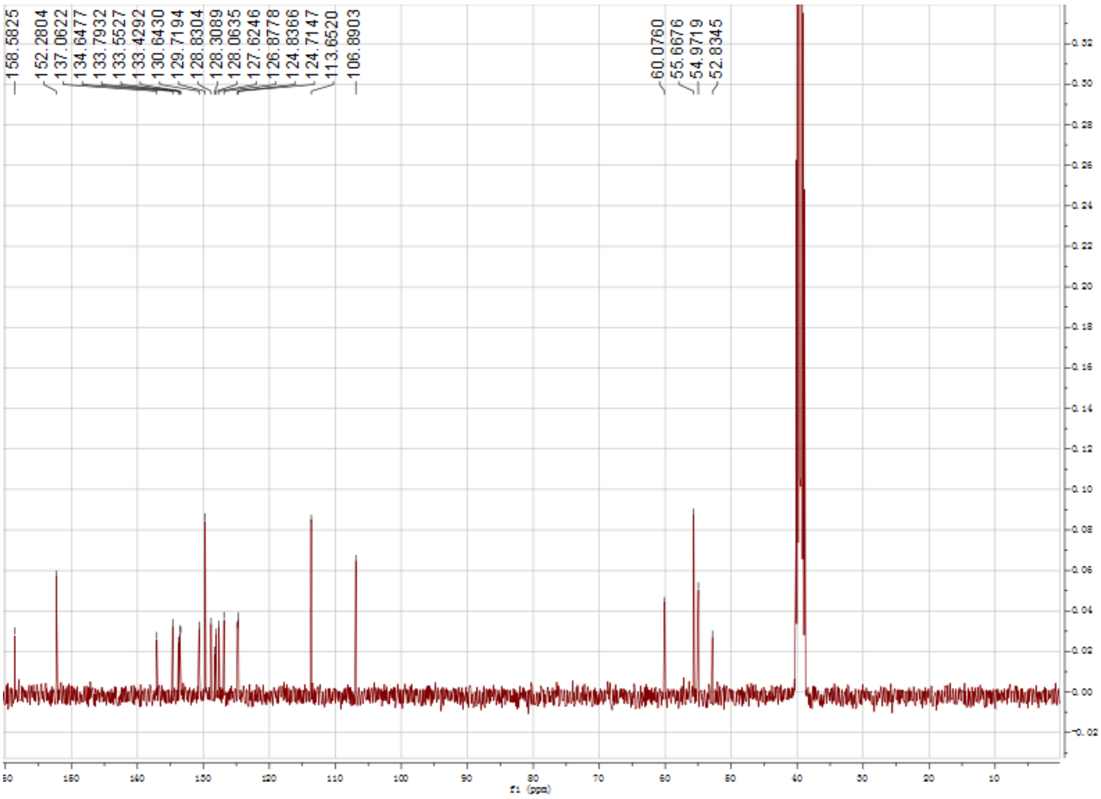

Figure S3. ^{13}C NMR of compound **5c**

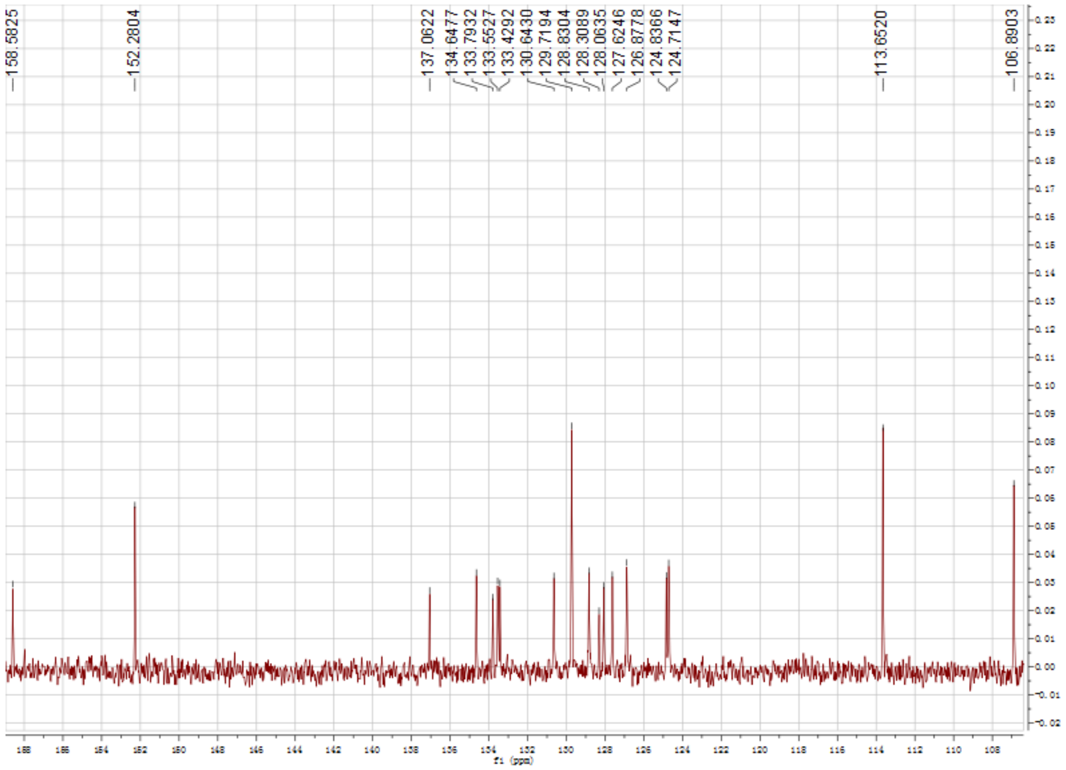

Figure S4. ^{13}C NMR of compound **5c**

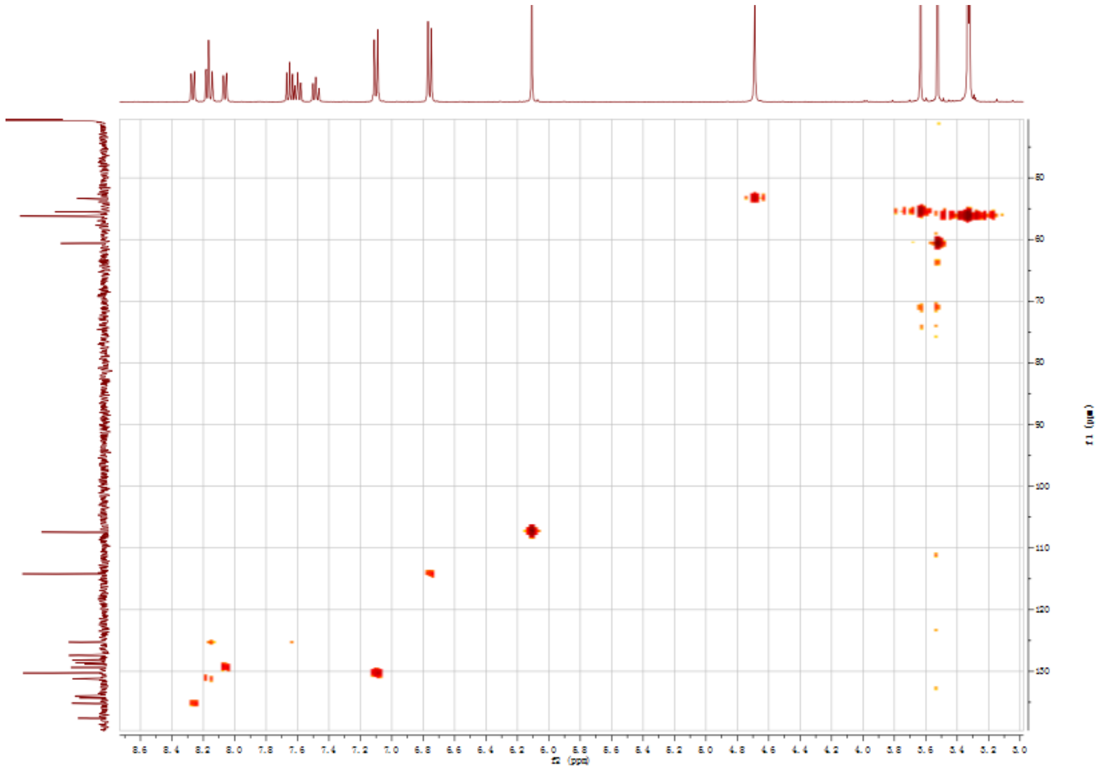

Figure S5. HMQC of compound **5c**

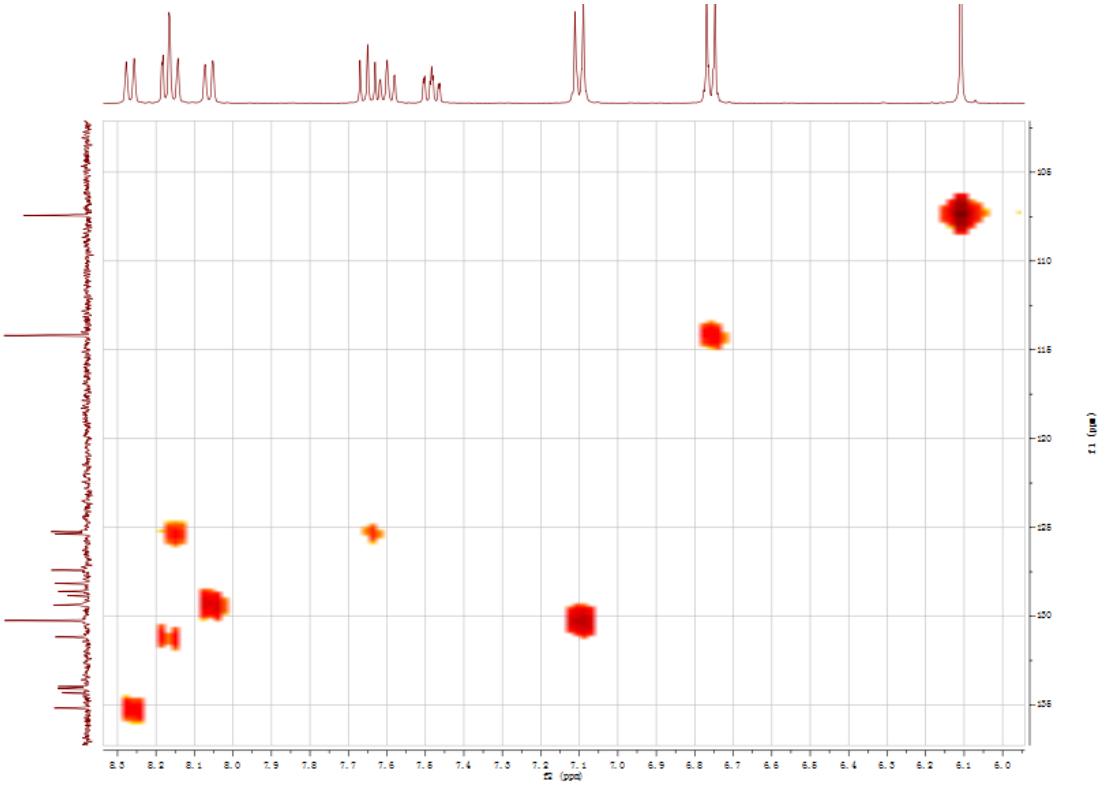

Figure S6. HMQC of compound **5c**

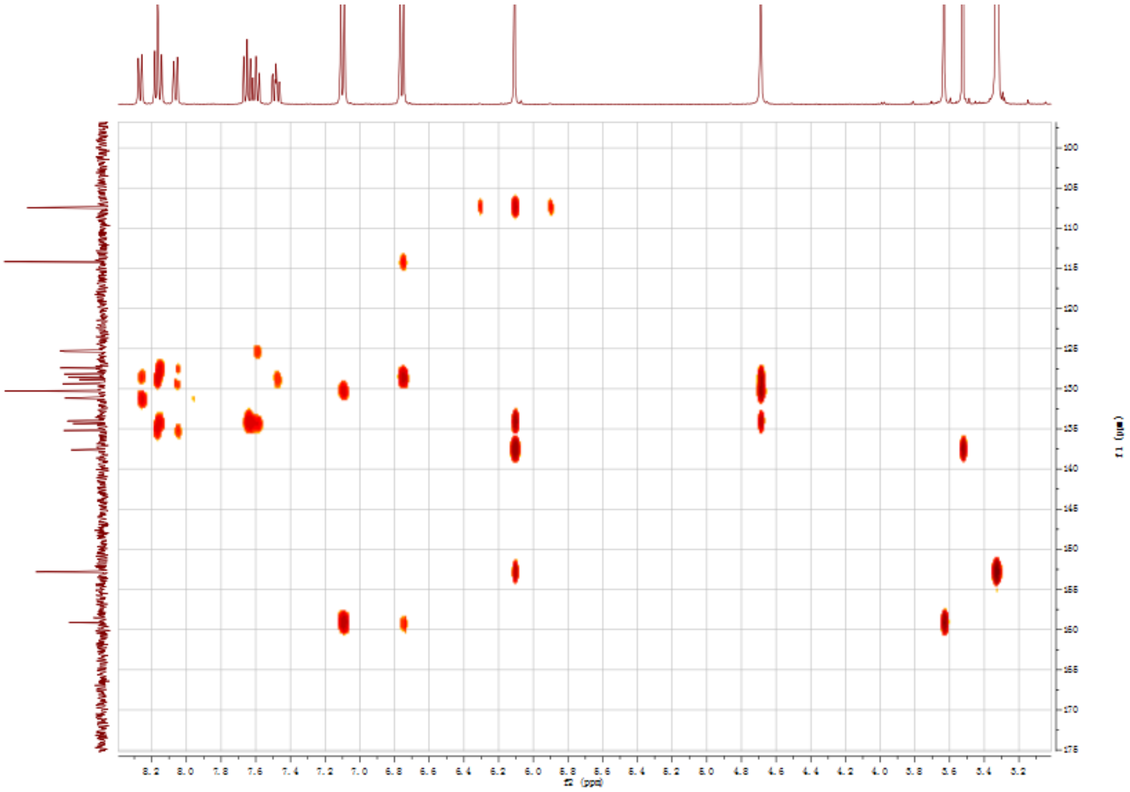

Figure S7. HMBC of compound **5c**

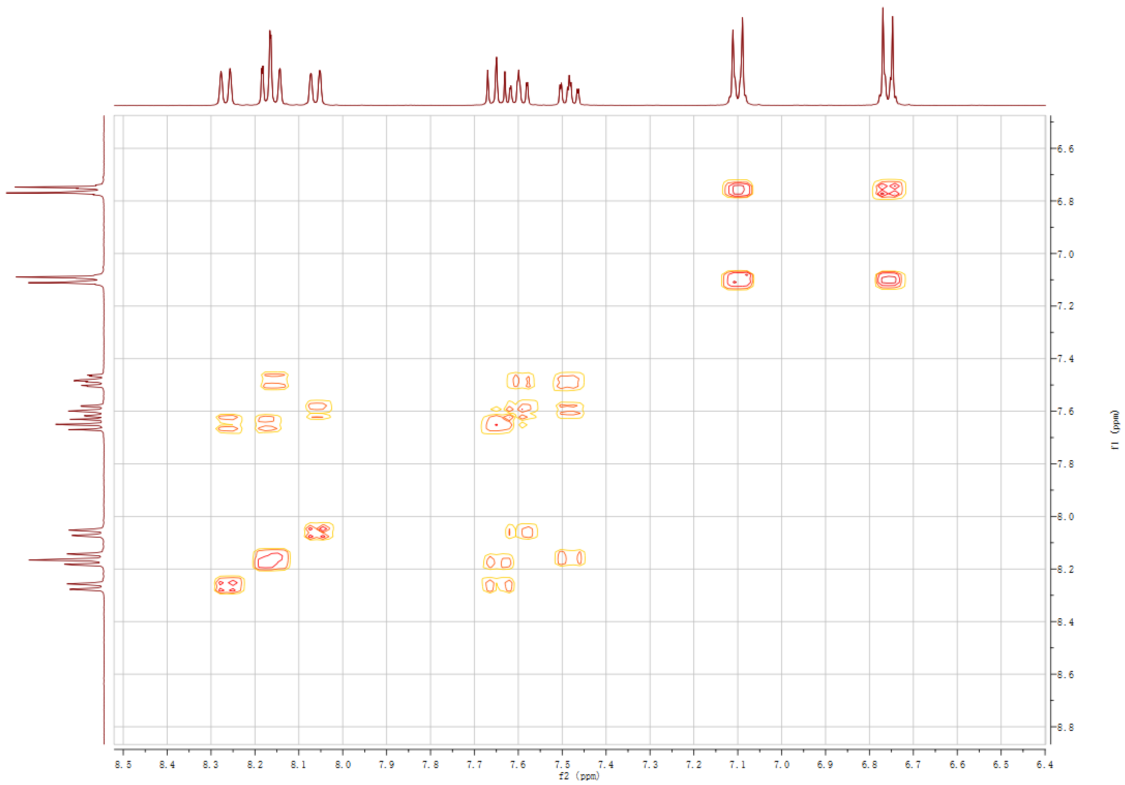

Figure S8. COSY of compound **5c**

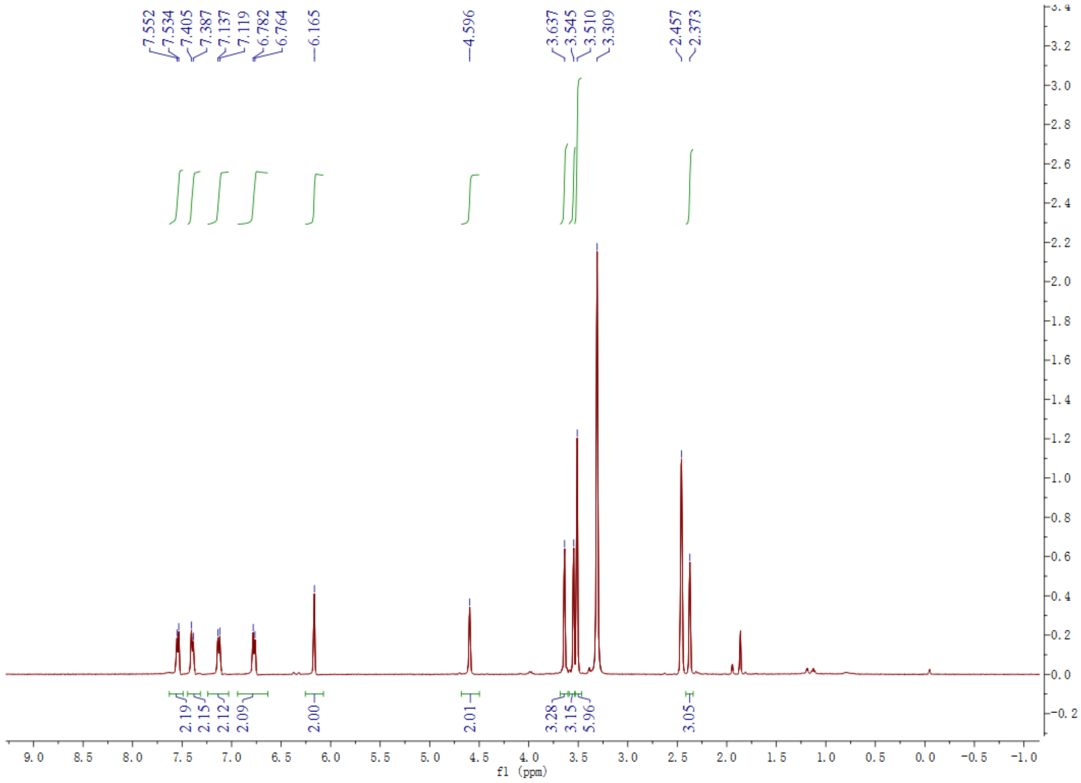

Figure S9. ^{1}H NMR of compound **5a**

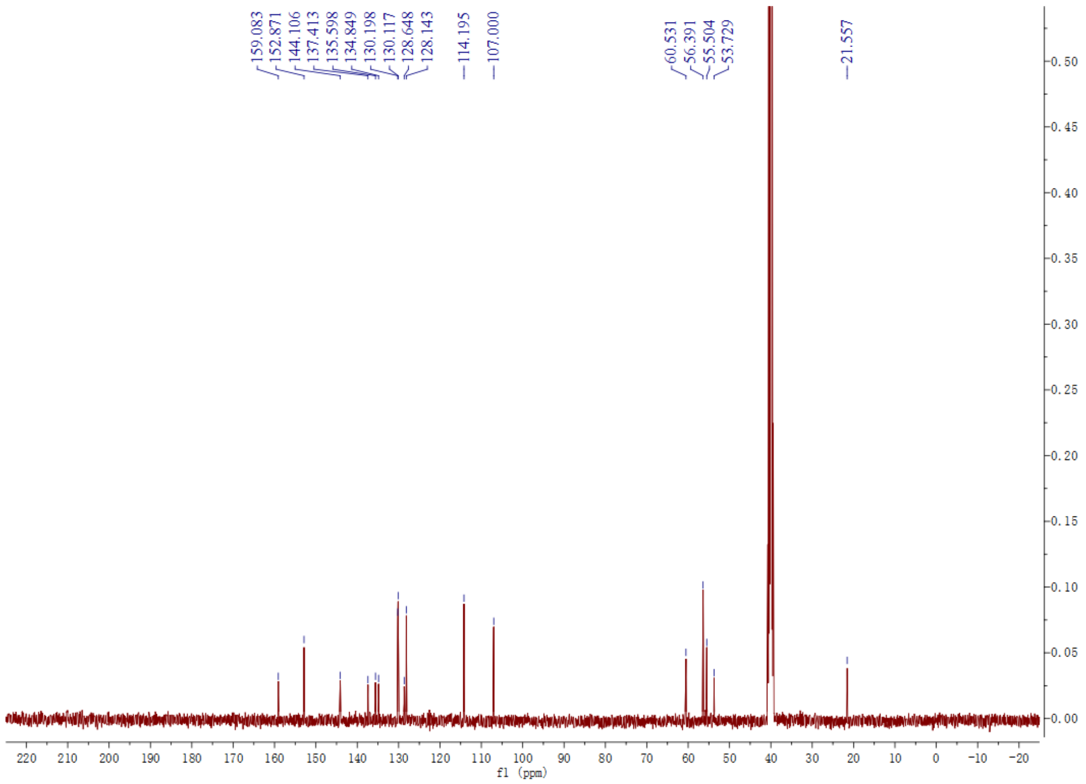

Figure S10. ^{13}C NMR of compound **5a**

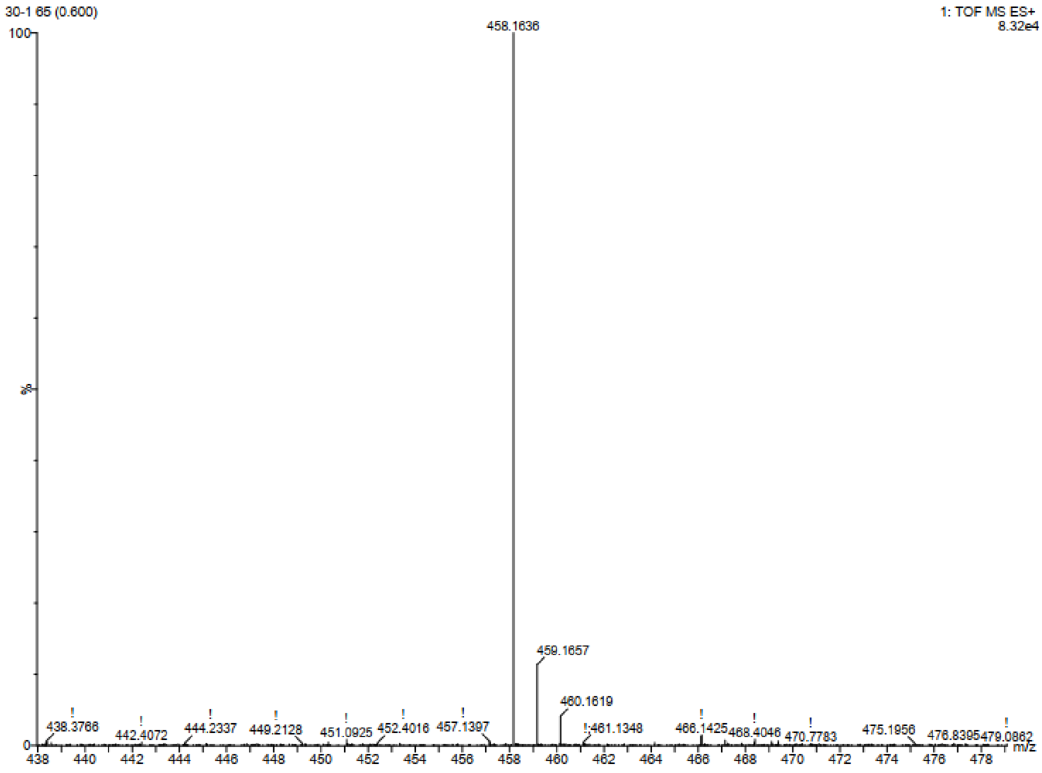

Figure S11. HRMS of compound **5a**

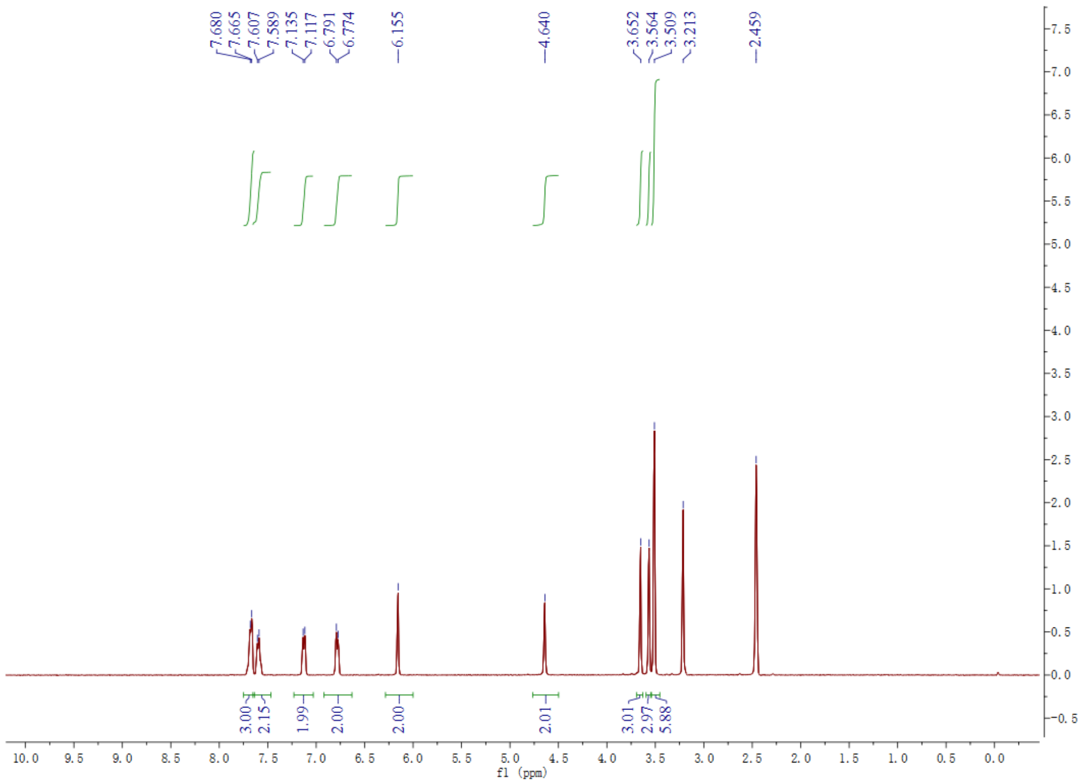

Figure S12. ^{1}H NMR of compound **5**b

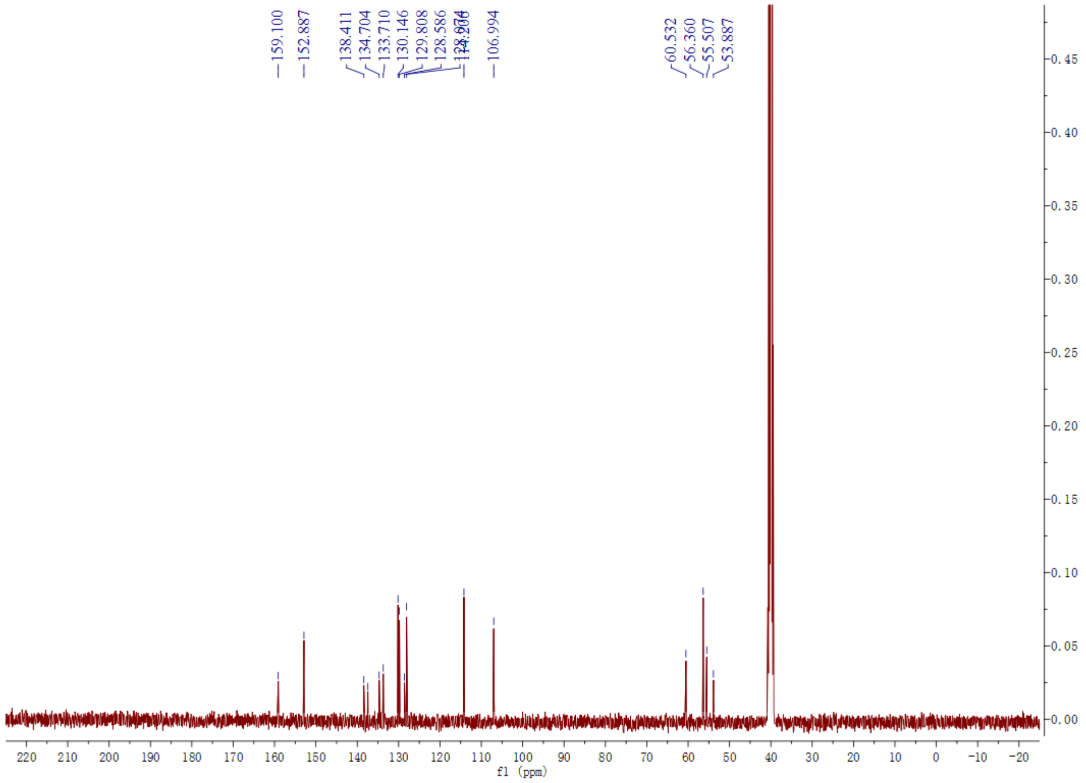

Figure S13. ^{13}C NMR of compound **5b**

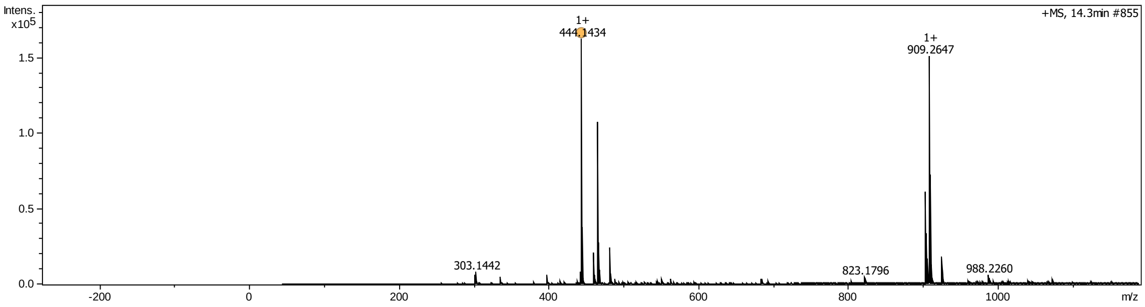

Figure S14. HRMS of compound **5b**

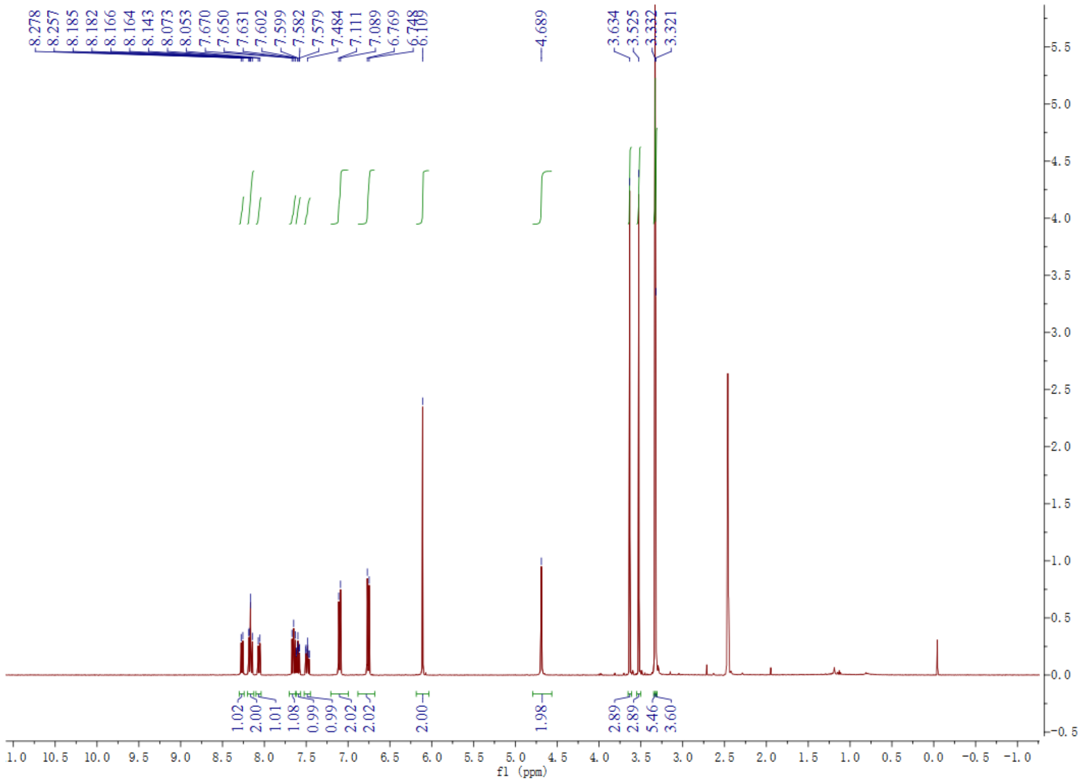

Figure S15. ^{1}H NMR of compound **5c**

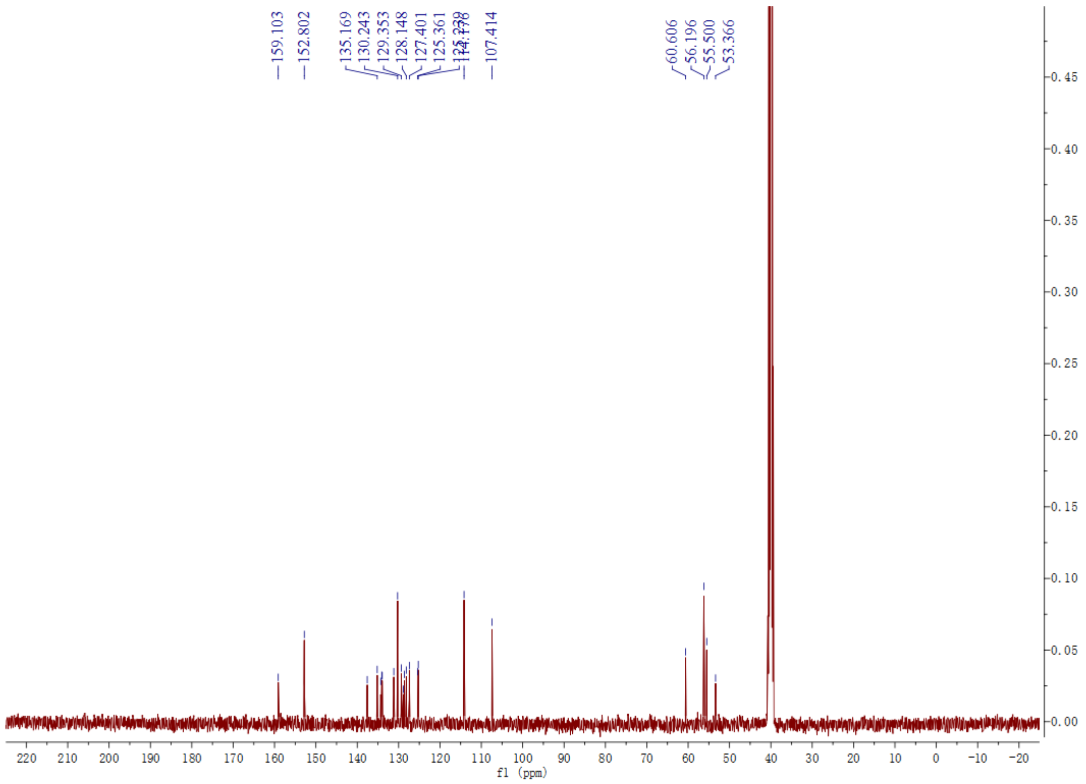

Figure S16. ^{13}C NMR of compound **5c**

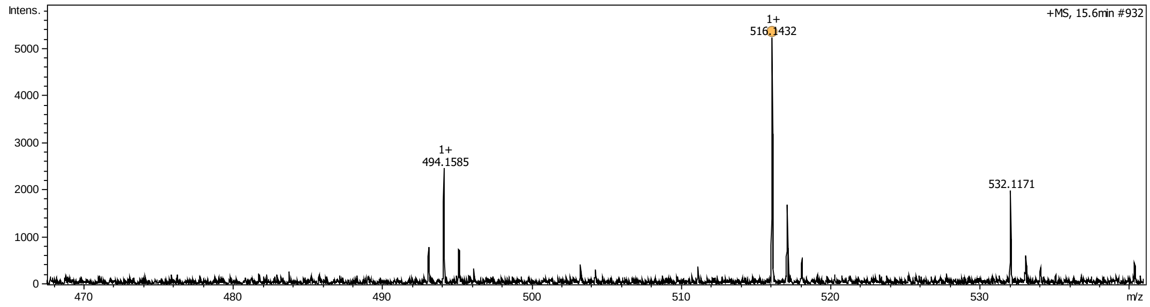

Figure S17. HRMS of compound **5c**

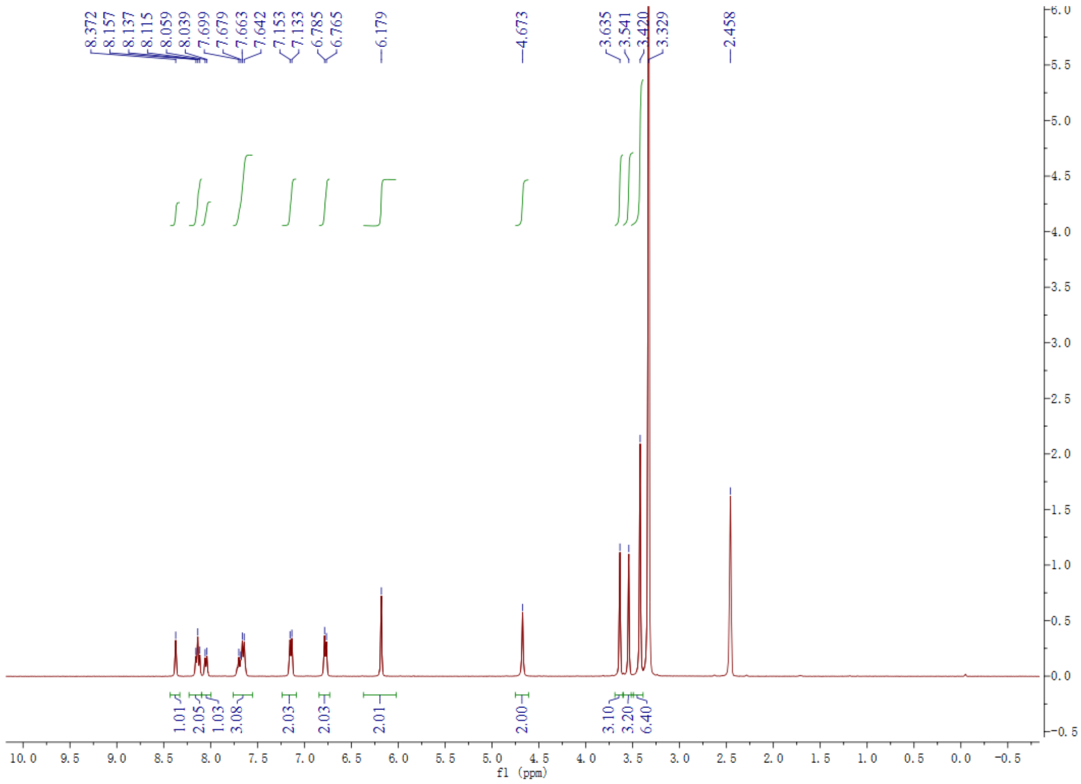

Figure S18. ^{1}H NMR of compound **5d**

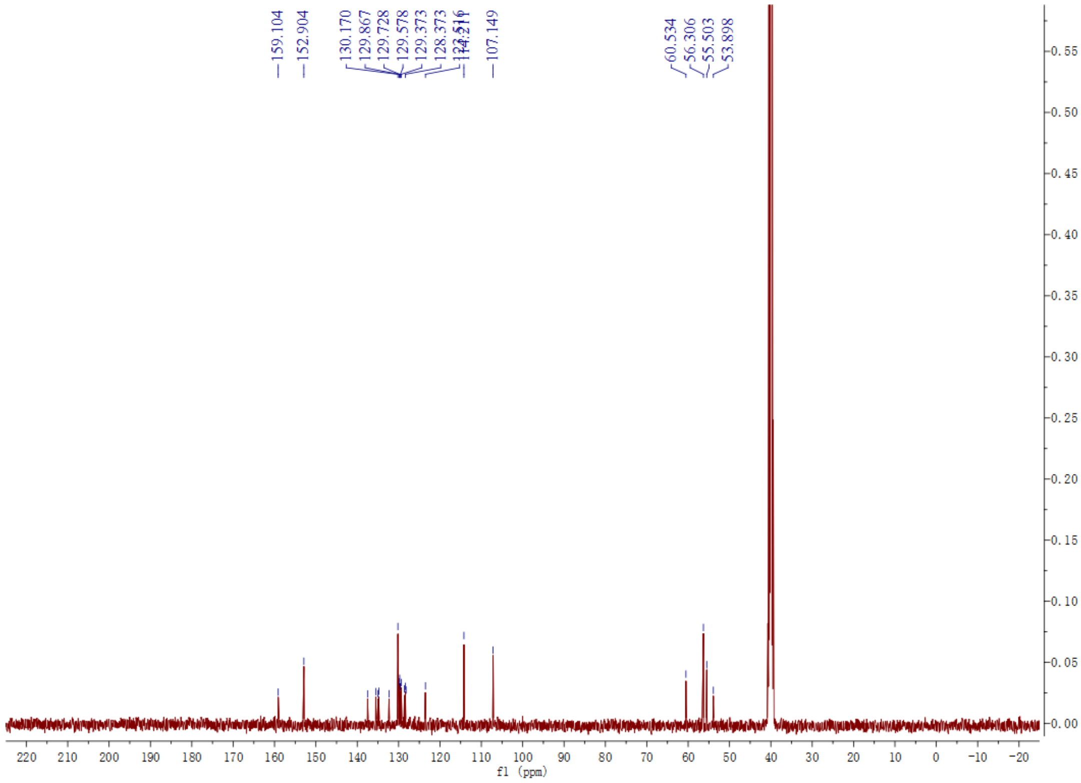

Figure S19. ^{13}C NMR of compound **5d**

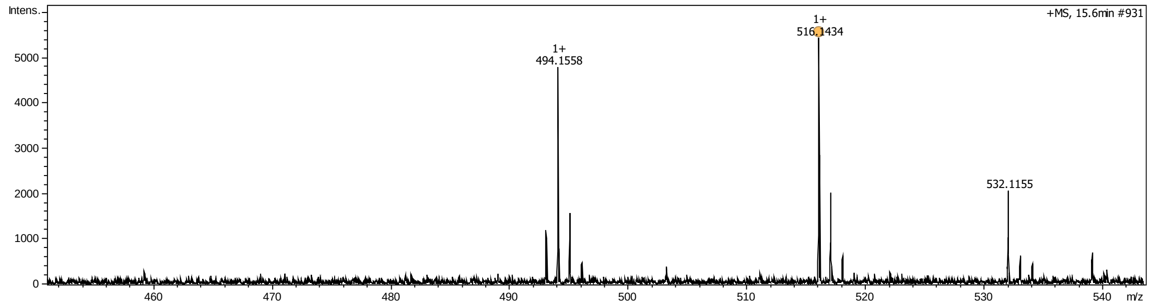

Figure S20. HRMS of compound **5d**

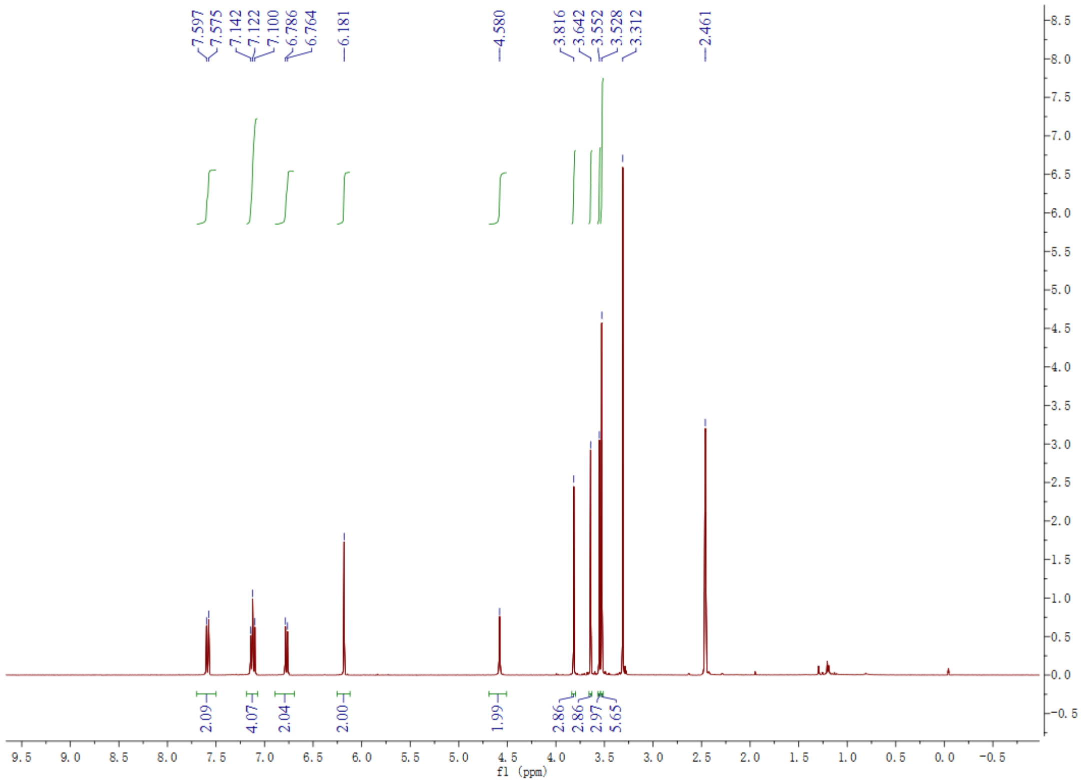

Figure S21. ^{1}H NMR of compound **5e**

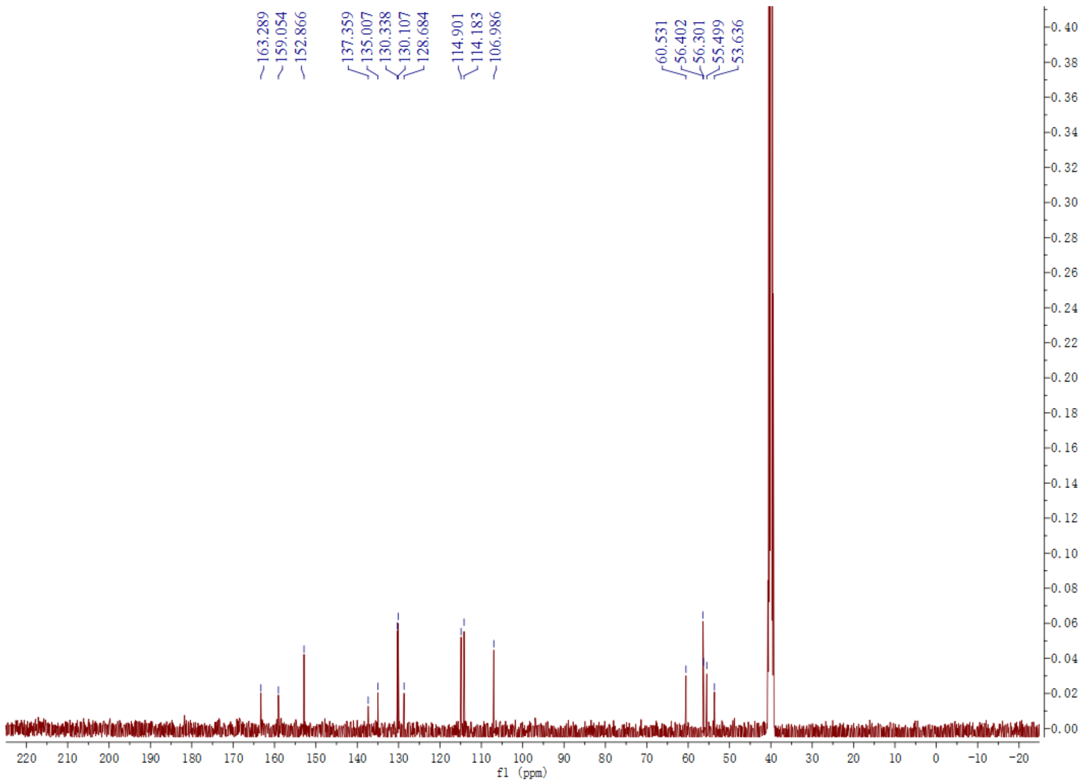

Figure S22. ^{13}C NMR of compound **5e**

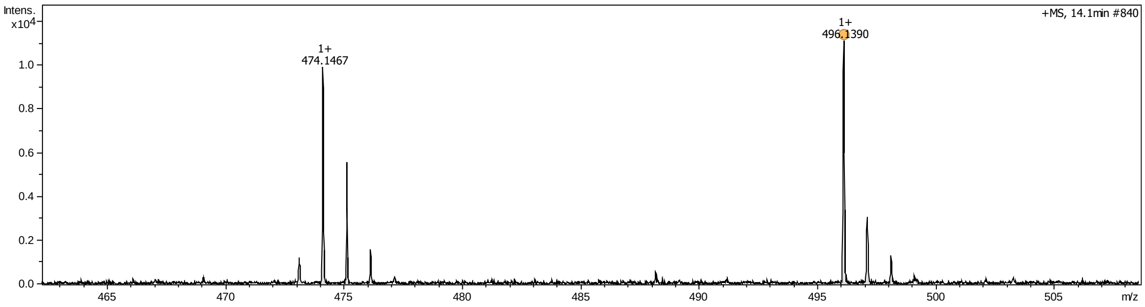

Figure S23. HRMS of compound **5e**

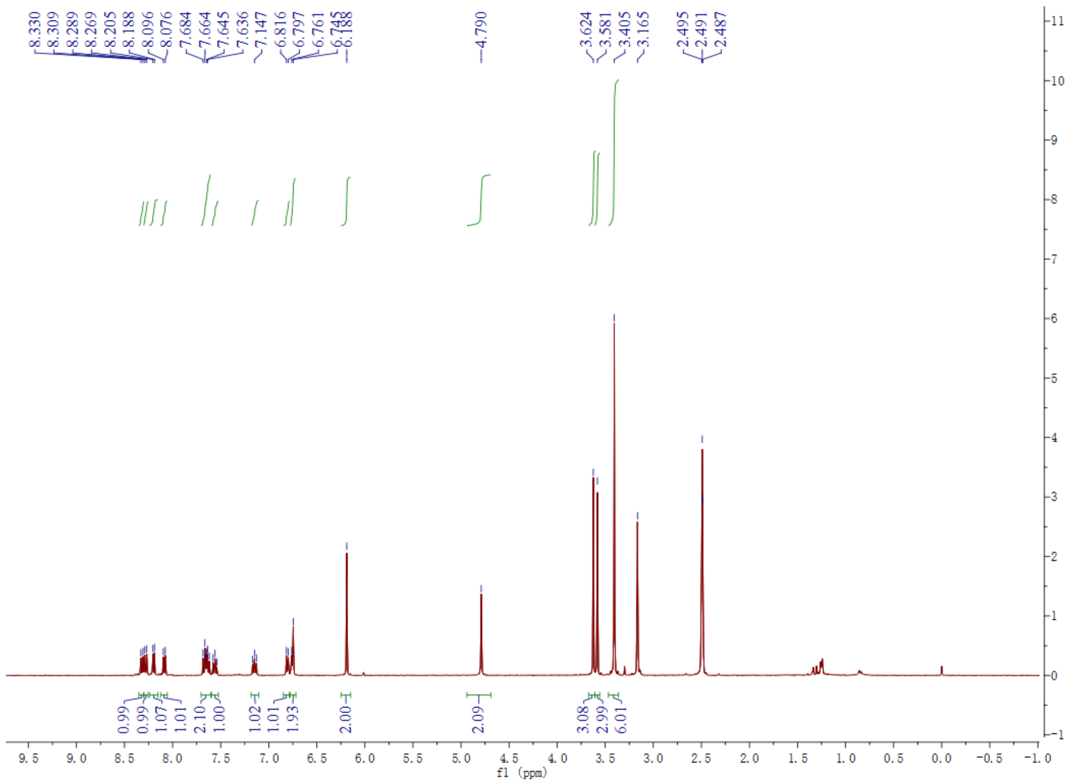

Figure S24. ^{1}H NMR of compound **8a**

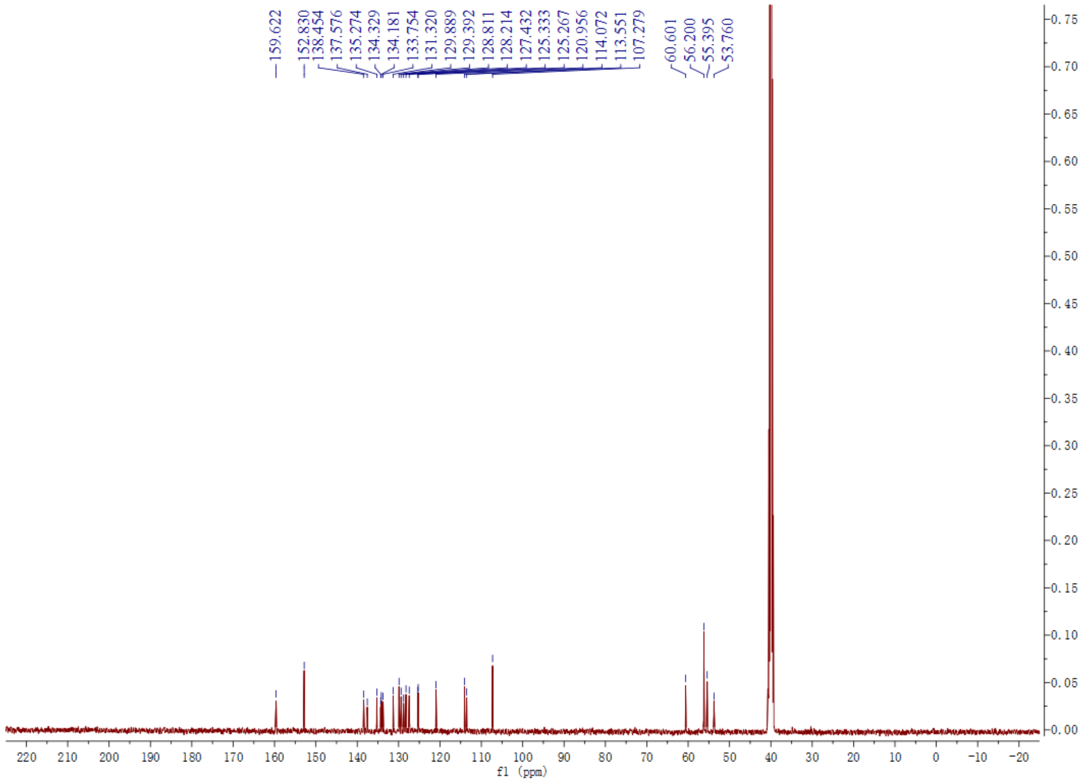

Figure S25. ^{13}C NMR of compound **8a**

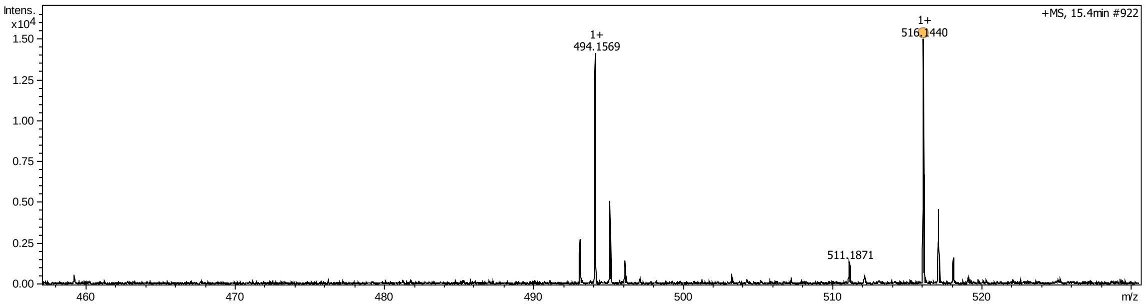

Figure S26. HRMS of compound **8a**

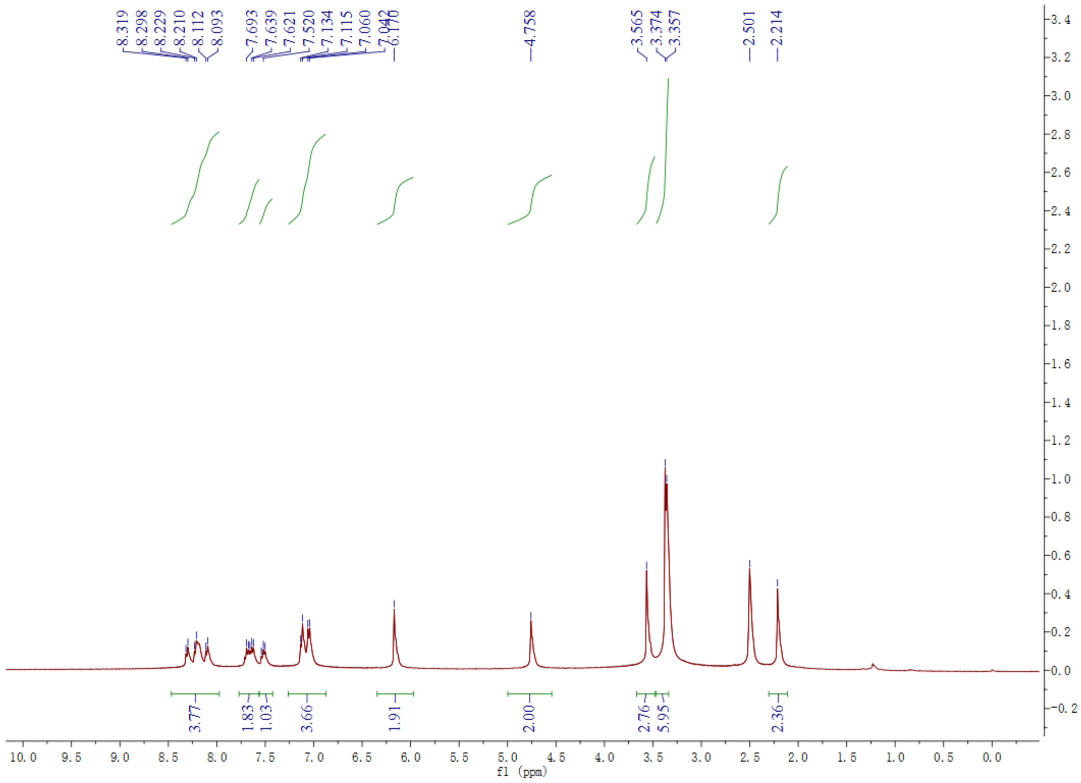

Figure S27. ^{1}H NMR of compound **8b**

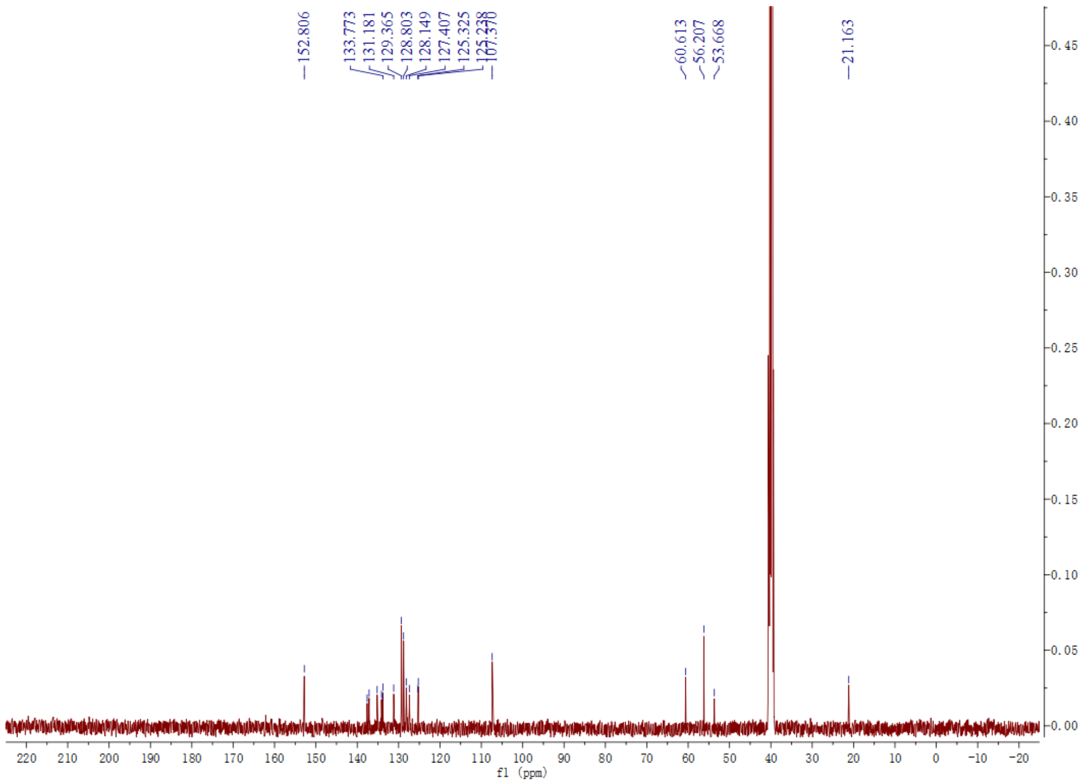

Figure S28. ^{13}C NMR of compound **8b**

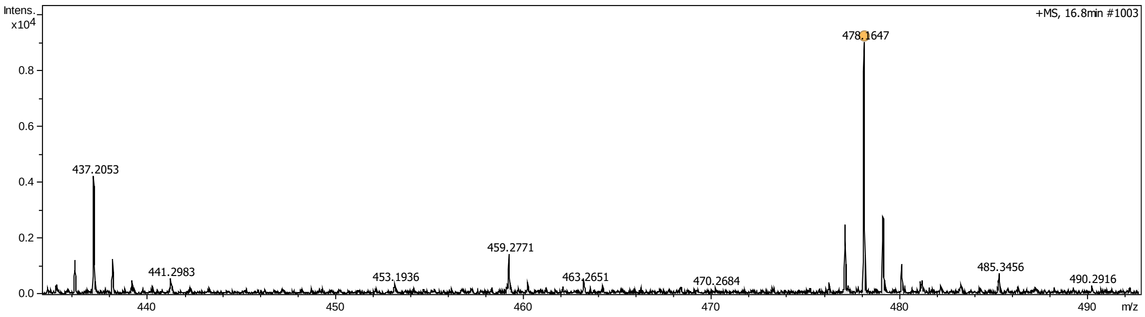

Figure S29. HRMS of compound **8b**

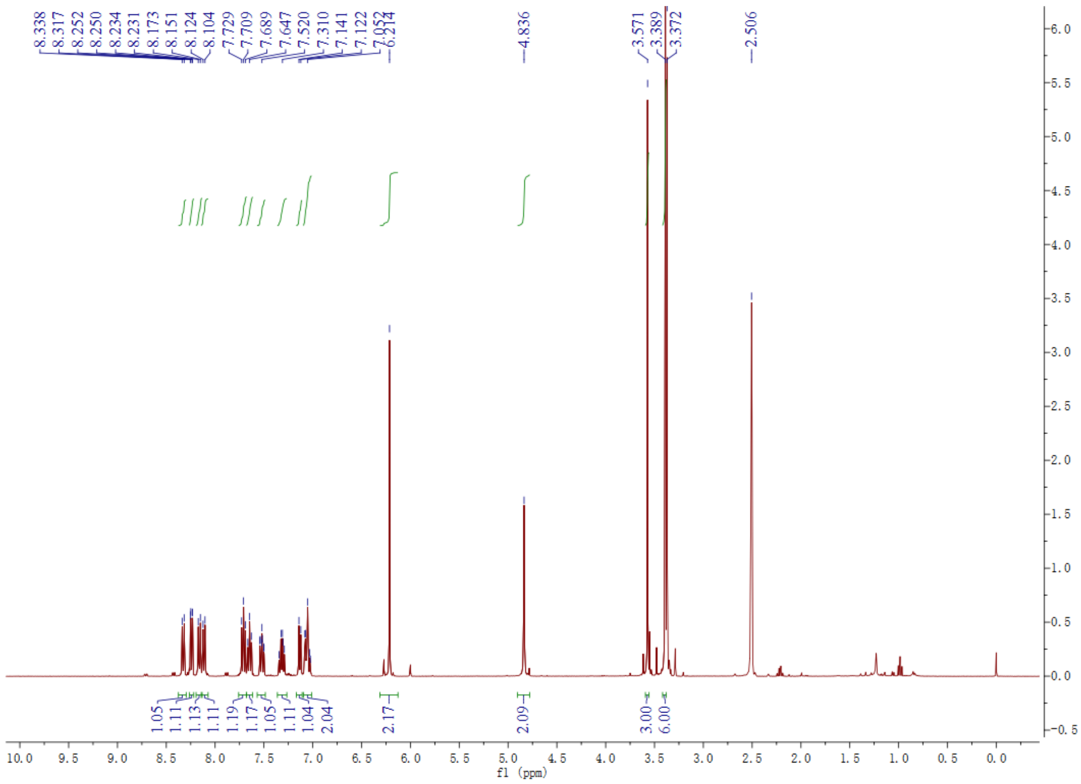

Figure S30. ^{1}H NMR of compound **8c**

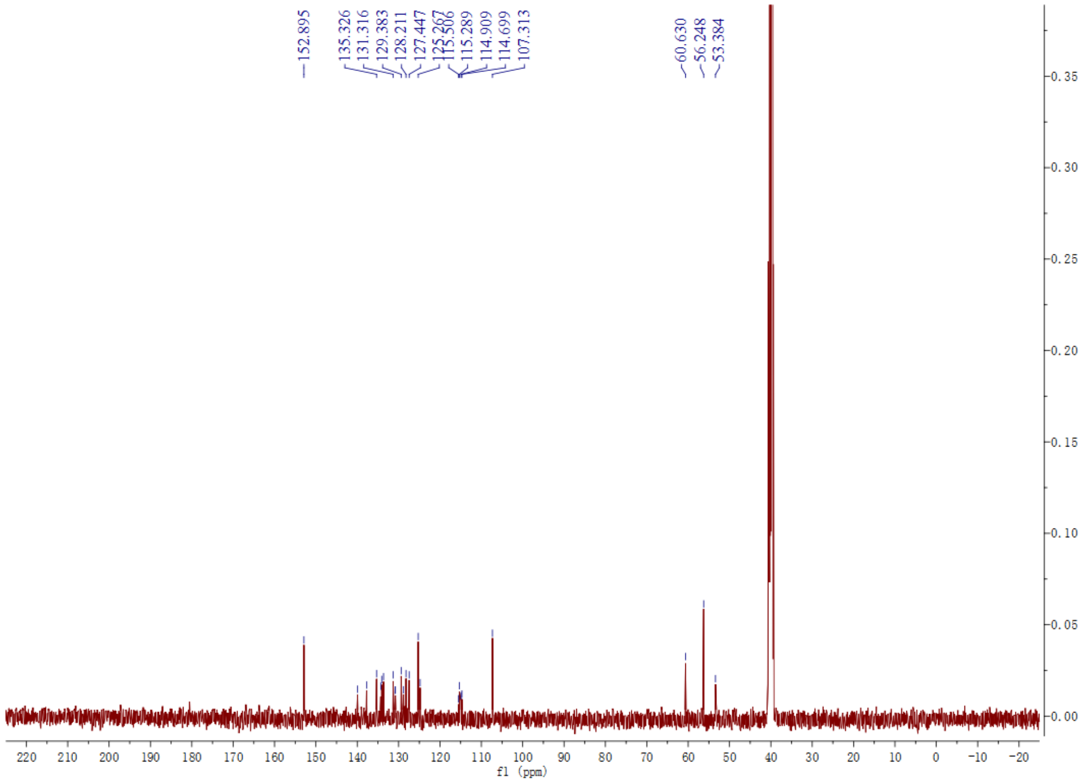

Figure S31. ^{13}C NMR of compound **8c**

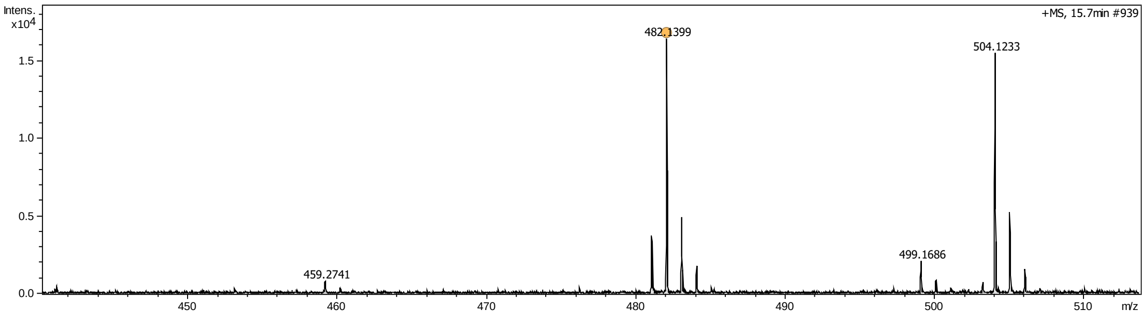

Figure S32. HRMS of compound **8c**

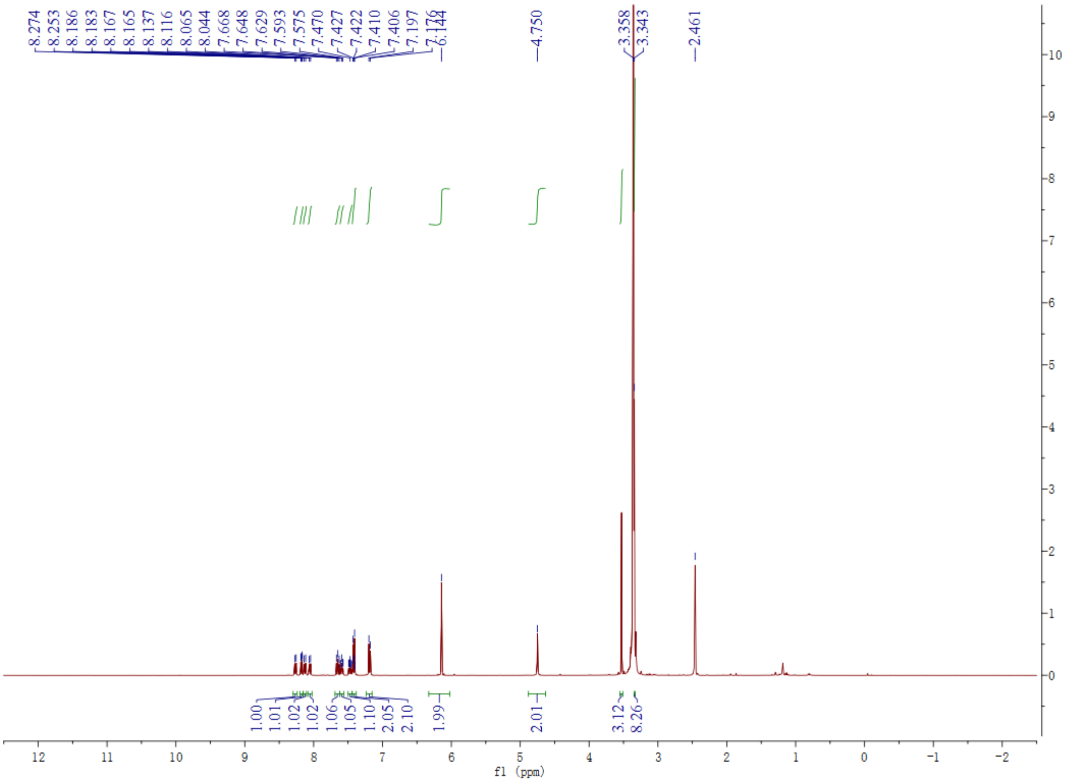

Figure S33. ^{1}H NMR of compound **8d**

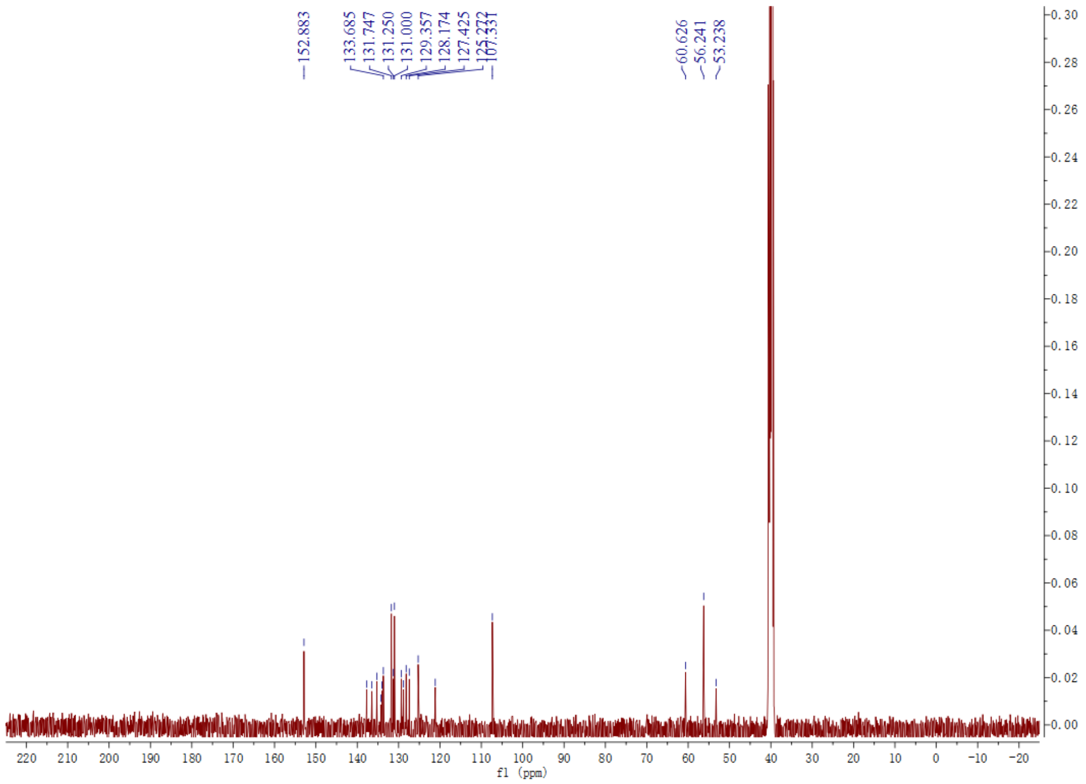

Figure S34. ^{13}C NMR of compound **8d**

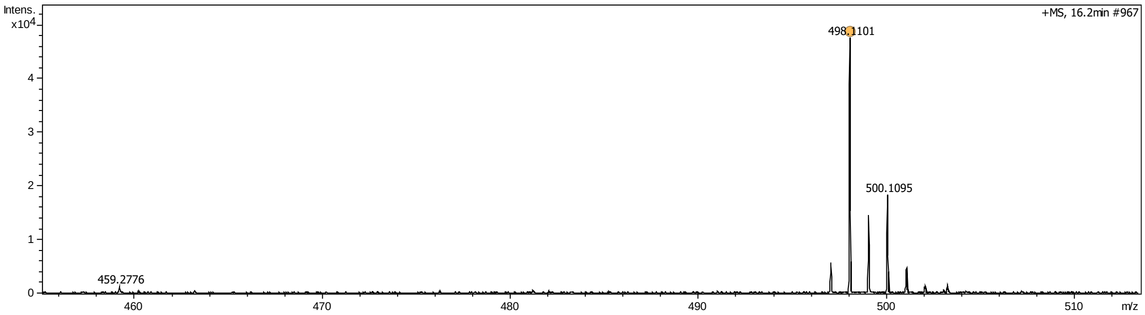

Figure S35. HRMS of compound **8d**

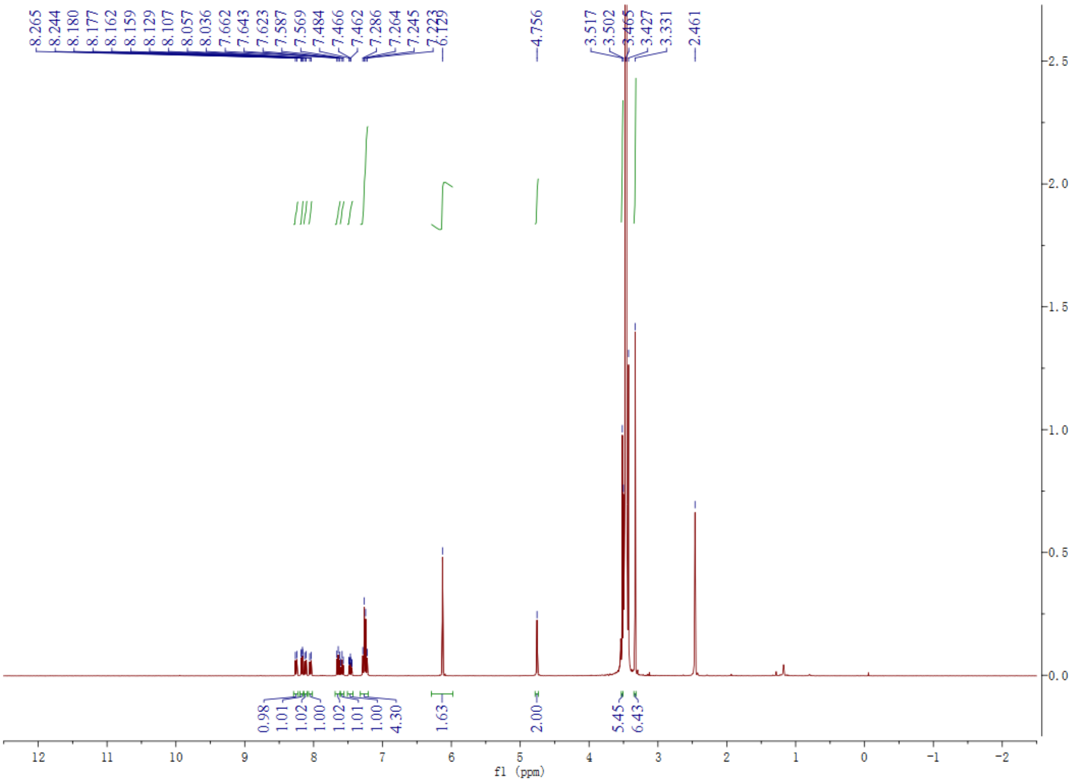

Figure S36. ^{1}H NMR of compound **8e**

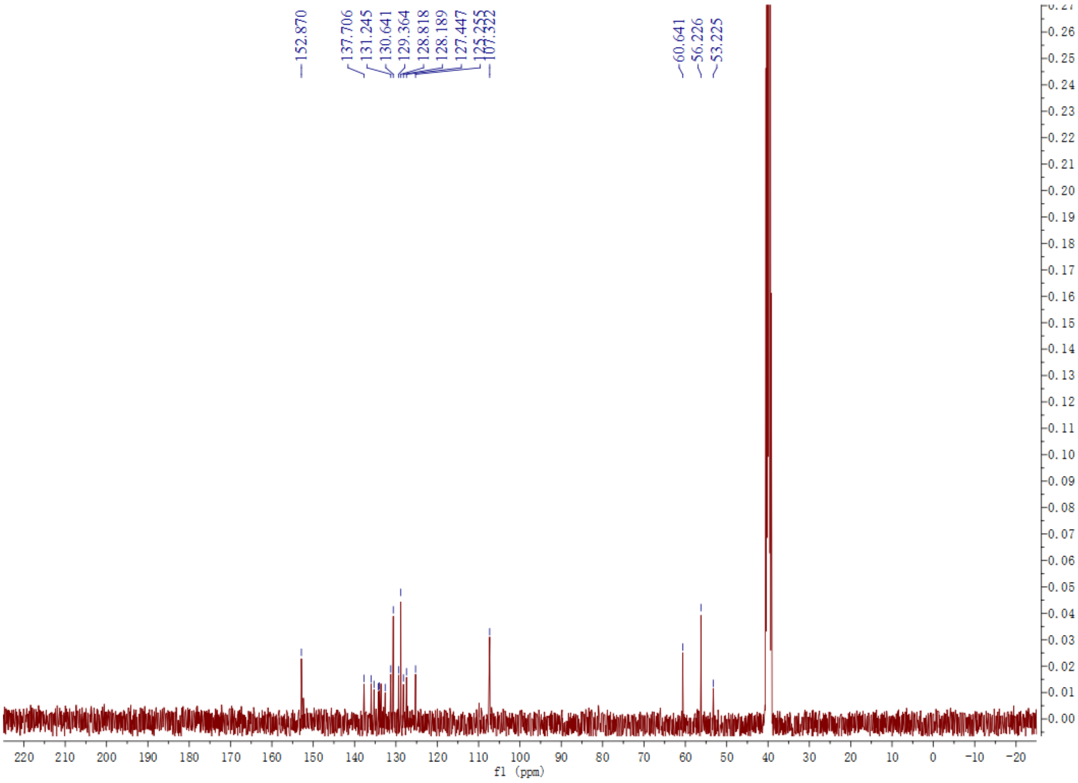

Figure S37. ^{13}C NMR of compound **8e**

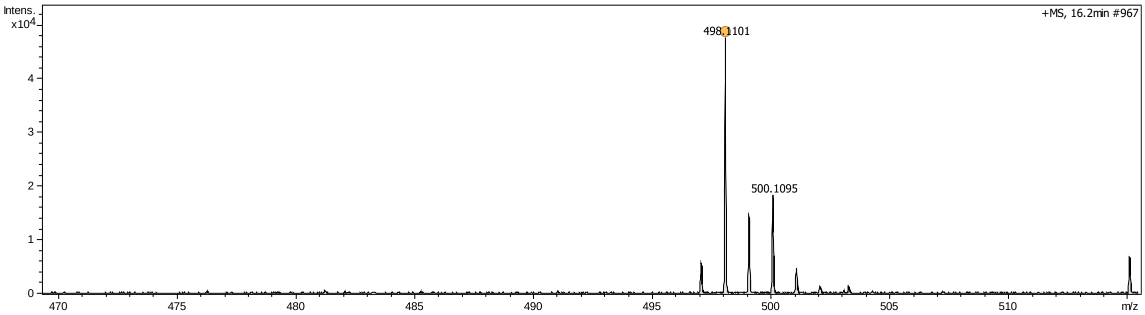

Figure S38. HRMS of compound **8e**

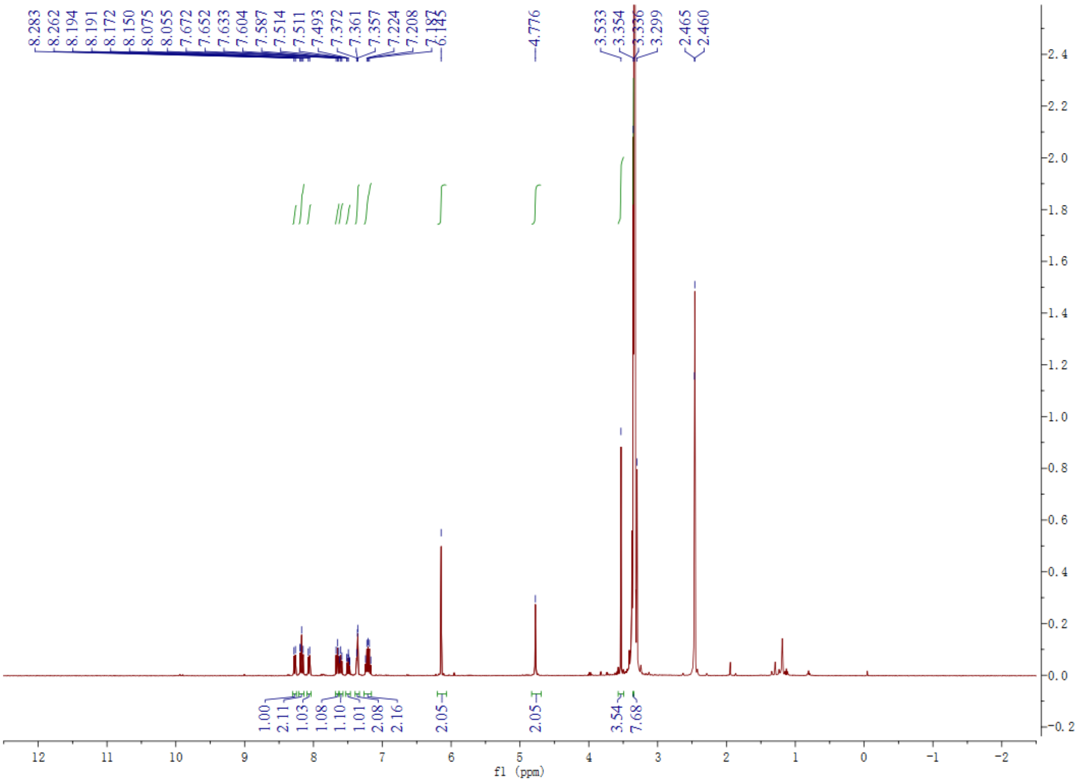

Figure S39. ^{1}H NMR of compound **8f**

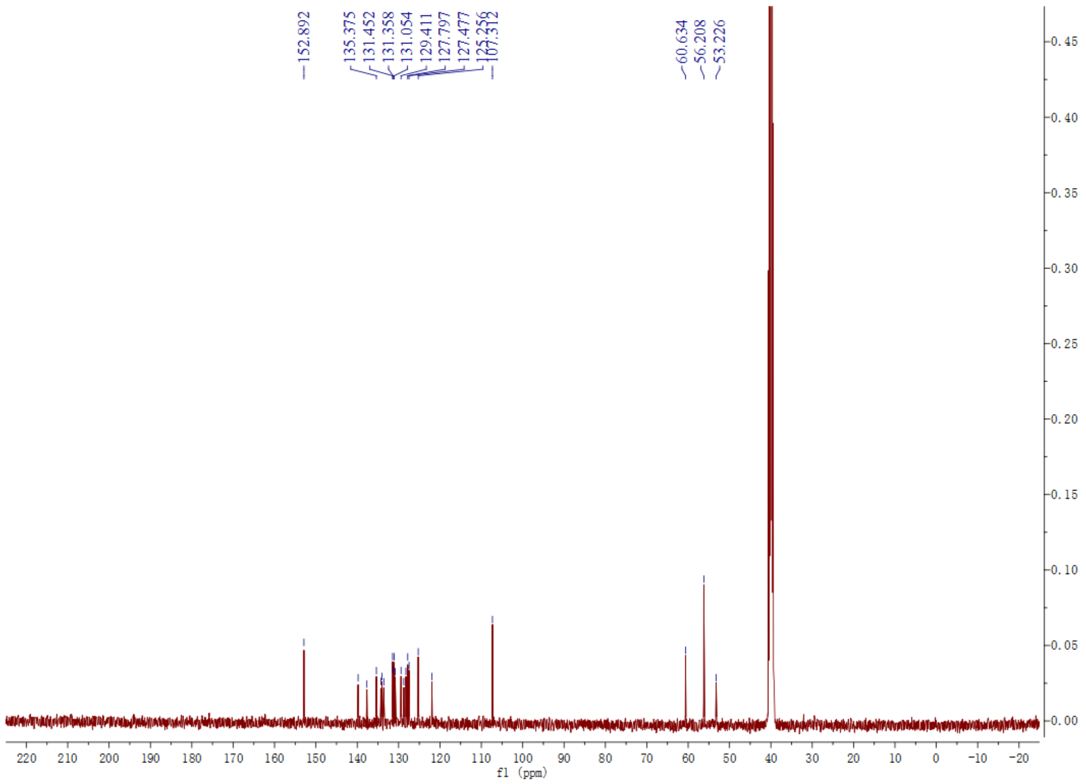

Figure S40. ^{13}C NMR of compound **8f**

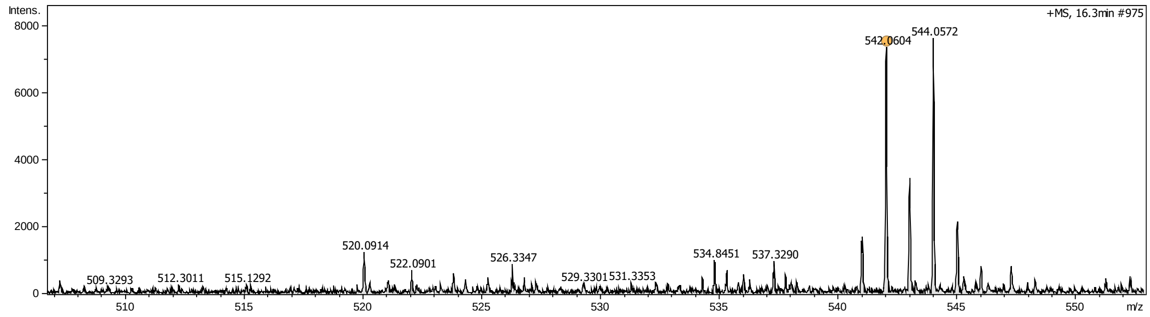

Figure S41. HRMS of compound **8f**

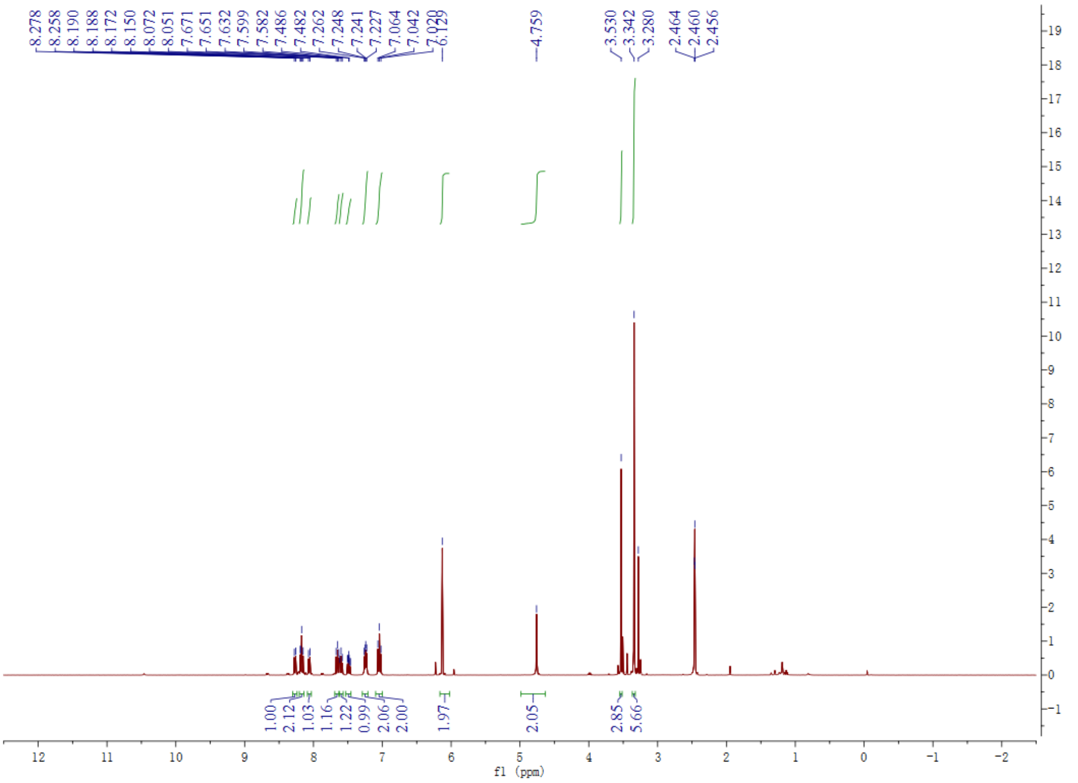

Figure S42. ^{1}H NMR of compound **8g**

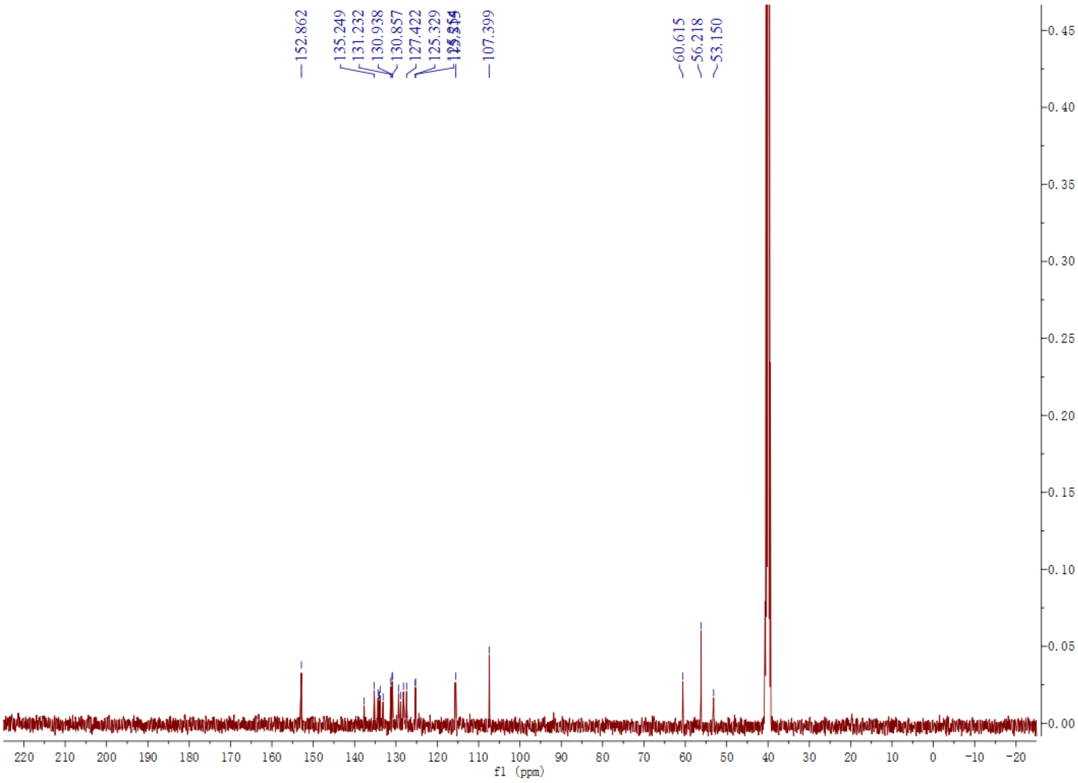

Figure S43. ^{13}C NMR of compound **8g**

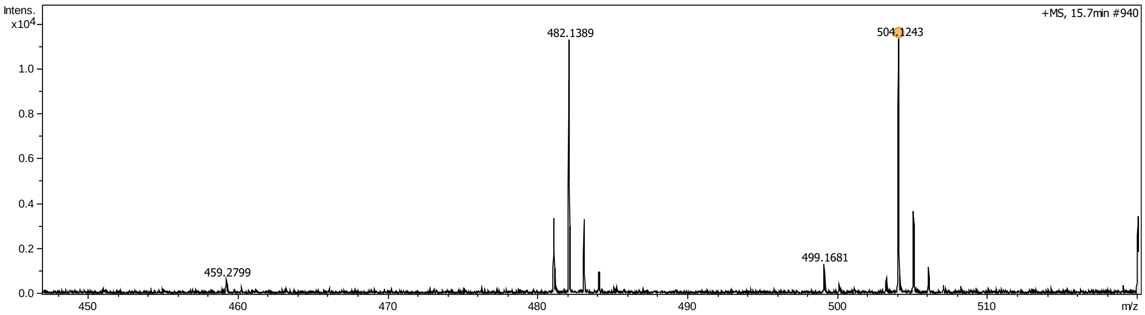

Figure S44. HRMS of compound **8g**

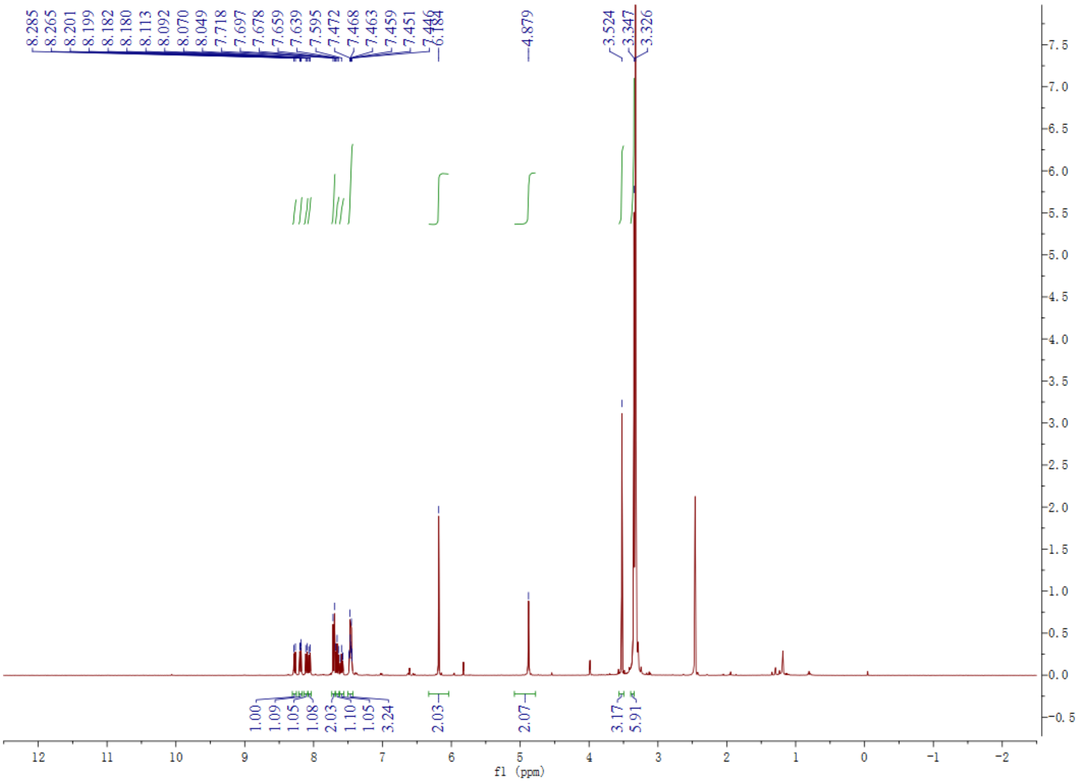

Figure S45. ^{1}H NMR of compound **8h**

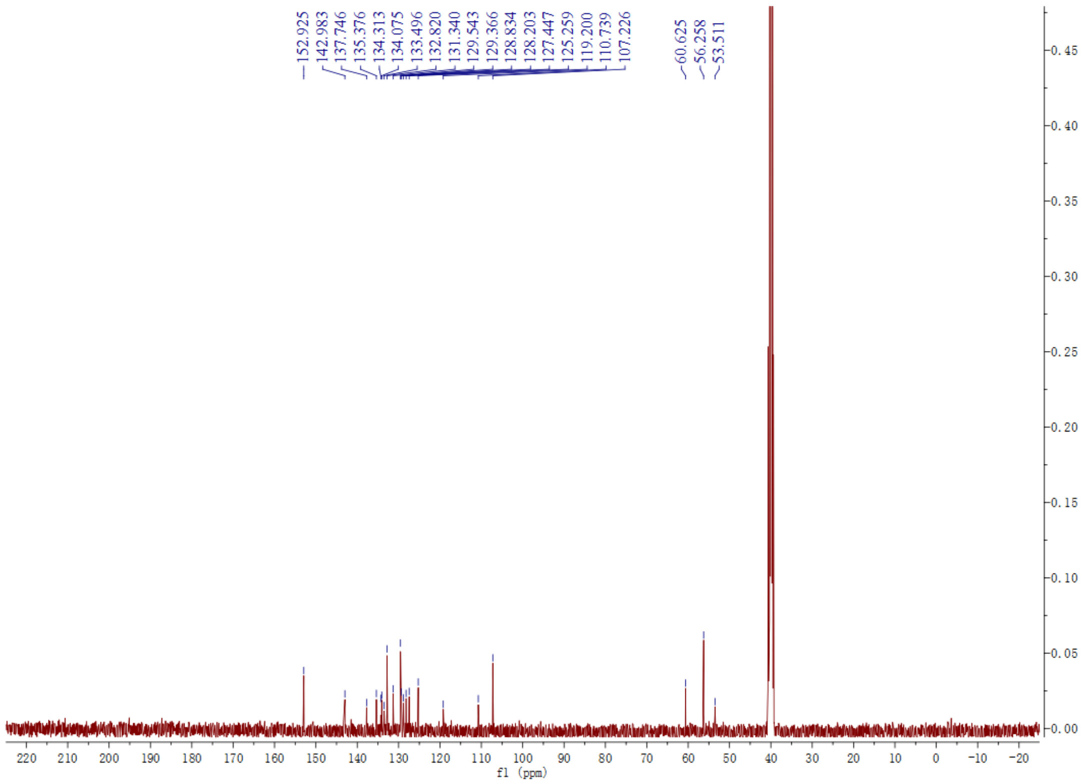

Figure S46. ^{13}C NMR of compound **8h**

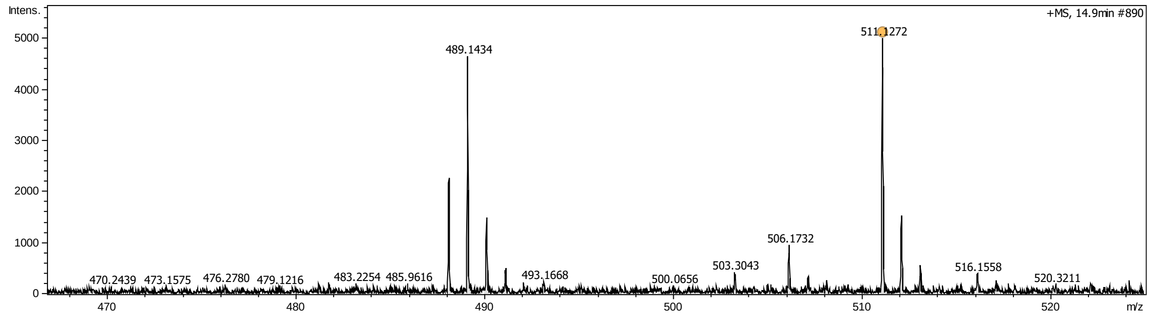

Figure S47. HRMS of compound **8h**

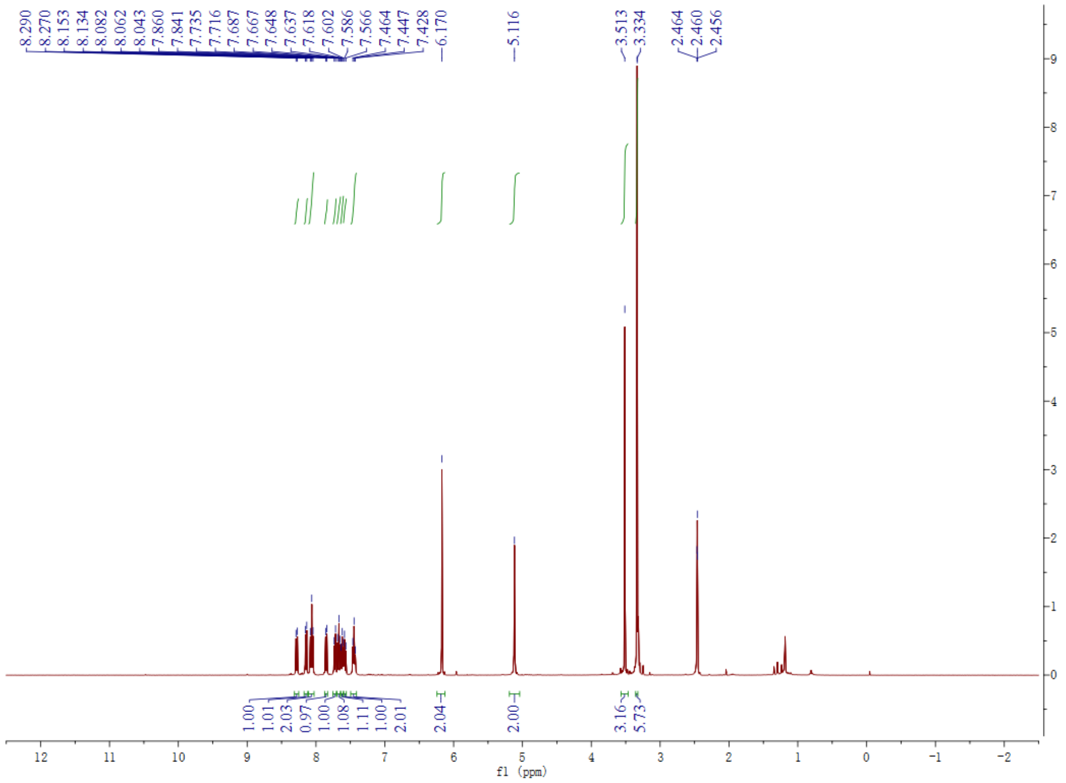

Figure S48. ^{1}H NMR of compound **8i**

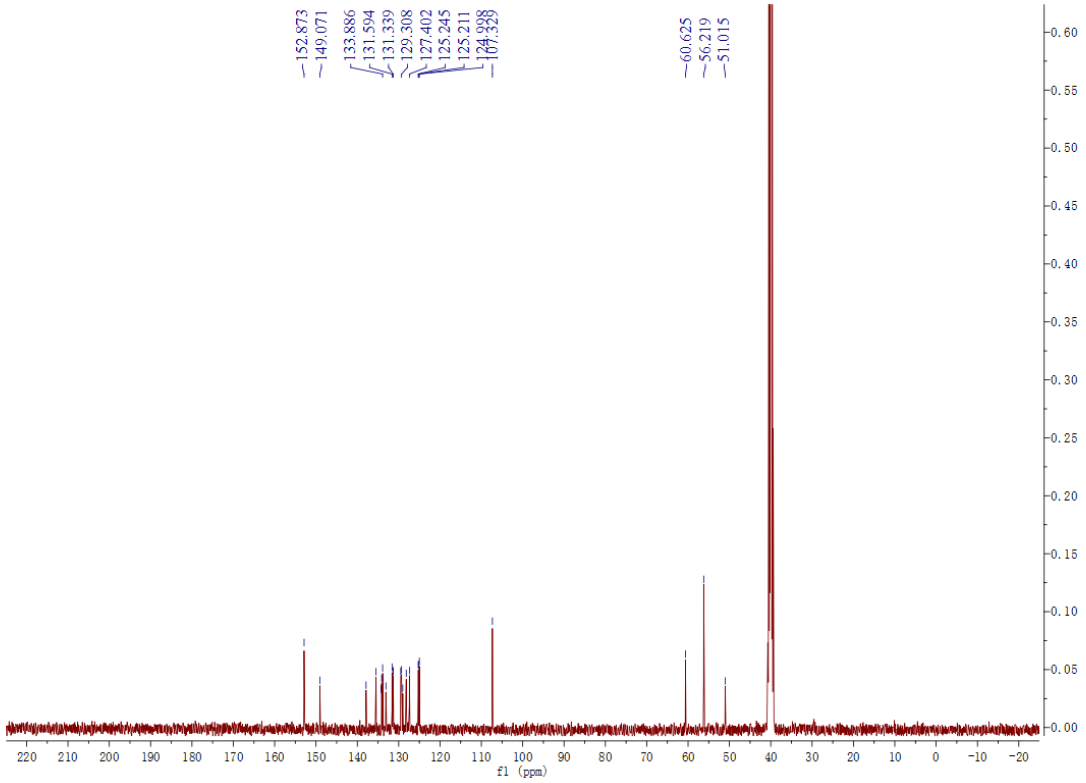

Figure S49. ^{13}C NMR of compound **8i**

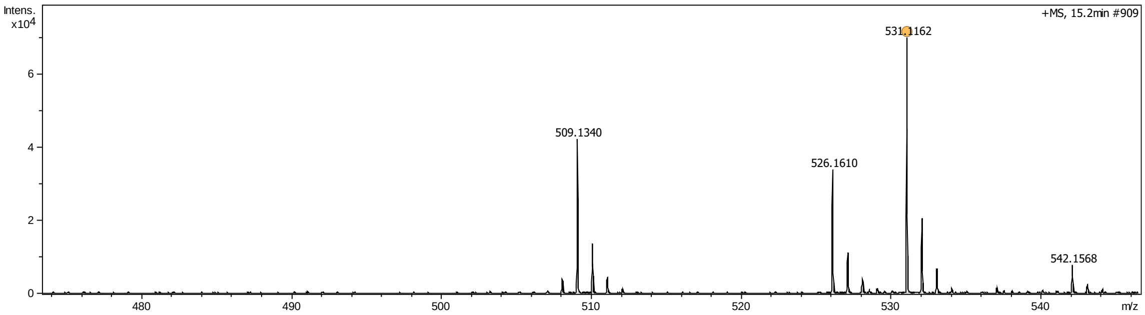

Figure S50. HRMS of compound **8i**

References

1. Pierce LC, Salomon-Ferrer R, Augusto F. de Oliveira C, et al. Routine access to millisecond time scale events with accelerated molecular dynamics. J Chem Theory Comput 2012;8:2997-3002.

2. Götz AW, Williamson MJ, Xu D, et al. Routine microsecond molecular dynamics simulations with amber on gpus. 1. Generalized born. Journal of chemical theory and computation 2012;8:1542-55.

3. Salomon-Ferrer R, Götz AW, Poole D, et al. Routine microsecond molecular dynamics simulations with amber on gpus. 2. Explicit solvent particle mesh ewald. Journal of Chemical Theory and Computation 2013;9:3878-88.

4. da Silva AWS, Vranken WF. Acpype-antechamber python parser interface. BMC Res Notes 2012;5:367.

5. Wang J, Wolf RM, Caldwell JW, et al. Development and testing of a general amber force field. J Comput Chem 2004;25:1157-74.

6. Wang J, Wang W, Kollman PA, et al. Automatic atom type and bond type perception in molecular mechanical calculations. J Mol Graph Model 2006;25:247-60.
